# Cortical Pyramidal and Parvalbumin Cells Exhibit Distinct Spatiotemporal Extracellular Electric Potentials

**DOI:** 10.1523/ENEURO.0265-22.2022

**Published:** 2022-12-06

**Authors:** Lior J. Sukman, Eran Stark

**Affiliations:** Sagol School of Neuroscience and Department of Physiology and Pharmacology, Sackler Faculty of Medicine, Tel Aviv University, Tel Aviv 6997801, Israel

**Keywords:** classification, electrophysiology, high-density arrays, hippocampus, interneurons, mouse

## Abstract

Brain circuits are composed of diverse cell types with distinct morphologies, connections, and distributions of ion channels. Modeling suggests that the spatial distribution of the extracellular voltage during a spike depends on cellular morphology, connectivity, and identity. However, experimental evidence from the intact brain is lacking. Here, we combined high-density recordings from hippocampal region CA1 and neocortex of freely moving mice with optogenetic tagging of parvalbumin-immunoreactive (PV) cells. We used ground truth tagging of the recorded pyramidal cells (PYR) and PV cells to construct binary classification models. Features derived from single-channel waveforms or from spike timing alone allowed near-perfect classification of PYR and PV cells. To determine whether there is unique information in the spatial distribution of the extracellular potentials, we removed all single-channel waveform information from the multichannel waveforms using an event-based delta-transformation. We found that spatiotemporal features derived from the transformed waveforms yield accurate classification. The extracellular analog of the spatial distribution of the initial depolarization phase provided the highest contribution to the spatially based prediction. Compared with PV cell spikes, PYR spikes exhibited higher spatial synchrony at the beginning of the extracellular spike and lower synchrony at the trough. The successful classification of PYR and PV cells based on purely spatial features provides direct experimental evidence that spikes of distinct cell types are associated with distinct spatial distributions of extracellular potentials.

## Significance Statement

It is not clear whether and how neuronal morphology, cell type, and synaptic inputs are mapped to the spatial distribution of the extracellular voltage during spikes. Here we show that spatial information alone allows accurate differentiation between pyramidal cells and parvalbumin-immunoreactive cells in neocortex and hippocampus of freely moving mice. The ability to distinguish cell types based on spatiotemporal properties of extracellular potentials suggests that neurons with distinct morphology, connectivity, and ion channel distributions create unique and learnable extracellular patterns. Further research may reveal whether unique spatial information is characteristic of other cell types.

## Introduction

Brain circuits are composed of different cell types with distinct roles in neuronal network dynamics (Tremblay et al., 2016; Tasic et al., 2018). Since the days of Brodmann (1909) and Ramón y Cajal (1909), it has been recognized that neurons in different brain regions, nuclei, and layers may have different morphologies (McKay and Turner, 2005; Ascoli et al., 2007; Clascá et al., 2012; Jones, 2012; Fujishima et al., 2018). Within a brain region, neurons that vary in the type of output (e.g., excitatory, inhibitory) and postsynaptic targets (e.g., somatic, dendritic, or axonal) exhibit distinct morphology (Markram et al., 2004; Klausberger and Somogyi, 2008; Kepecs and Fishell, 2014). Histologic and *in vitro* studies showed that the size, form, and orientation of the soma, the dendritic tree, and the axonal arbor all vary between cells that express different genes and neurochemical markers (Monyer and Markram, 2004; Zeisel et al., 2015; Zeng and Sanes, 2017).

In behaving animals, extracellular recording techniques allow simultaneous recording of electrical potentials generated by multiple neurons and sampling every neuron at several spatial locations (Buzsáki, 2004; Shobe et al., 2015; Jun et al., 2017; Hong and Lieber, 2019; Steinmetz et al., 2021). Multisite recordings with well defined electrode geometry open the door to blind cell type classification based on electrophysiological properties. The relation between morphology (structure) and neuronal cell type (function) is well established *in vitro* and using postmortem immunohistology (McCormick et al., 1985; Freund and Buzsáki, 1996; Somogyi and Klausberger, 2005). Extensive modeling work has been dedicated to understand the relationship between the spatial distribution of extracellular electrical potentials resulting from spikes and neuronal morphology (Rall, 1962; Gold et al., 2009; Einevoll et al., 2013). However, the relationship between the spatial distribution of extracellular electrical potentials and neuronal cell types in the intact brain remains unexplored.

To determine whether spikes of different cell types give rise to distinct distributions of extracellular potentials, we focus here on pyramidal cells (PYR) and parvalbumin-immunoreactive (PV) cells in neocortex and hippocampal region CA1. PYR have pyramid-shaped somata and vertically oriented, apical and basal (polar) dendritic trees (Spruston, 2008). In contrast, PV (mainly basket) cells have less polarized dendritic trees and axonal arbors that extend horizontally (Maccaferri et al., 2000; Pawelzik et al., 2002; Ganter et al., 2004). Furthermore, PYR and PV cells exhibit distinct spatial profiles of ion channels. While similar somatodendritic gradients are observed for Na^+^ channels, K^+^ channels exhibit a steeper decreasing gradient along dendrites farther from the soma in PYR, compared with PV cells (Magee and Johnston, 1995; Johnston et al., 2000; Hu et al., 2010). To go beyond descriptive structure–function relations, we hypothesized that PYR and PV cells could be classified based solely on spatial information acquired from freely moving mice using high-density probes. Previously, classification of PYR and interneurons in neocortex and hippocampus was based on waveform features (Henze et al., 2002; Barthó et al., 2004; Cardin et al., 2009; Stark et al., 2013; Mendoza et al., 2016; Yu et al., 2019), firing patterns (Taira and Georgopoulos, 1993; Kobayashi et al., 2019; Troullinou et al., 2020), or combinations thereof (Csicsvari et al., 1998; Frank et al., 2001; Viskontas et al., 2007). However, cell type classification based on spatial features per se was never attempted.

Here, we used connectivity-based and optical tagging to establish a dataset of labeled PYR and PV cells from neocortex and CA1 of freely moving mice. To tune the classification procedure and determine baseline performance, we first created classification models that used features based on single-channel waveforms or on spike timing. Using a chunking-based data augmentation method, the models achieve near-perfect performance. Next, we devised an event-based δ-transformation method to conserve only purely spatial information and derived spatial features from the multichannel recordings. Models trained on spatial features derived from the transformed waveforms yield accurate classification. The findings suggest that differences between PYR and PV cell neuronal morphology, connectivity, and ion channel distributions are reflected in the extracellular potentials in a consistent manner.

## Materials and Methods

### Experimental design

The dataset used in this study has been previously analyzed (Stark et al., 2013).

#### Experimental animals

Seven PV::ChR2 male mice, generated by crossing homozygous male Ai32 mice (catalog #012569, The Jackson Laboratory) with homozygous female PV-Cre mice (catalog #008069, The Jackson Laboratory), were used for chronic recordings. The animals and data were used for the work by Stark et al. (2013). All animal handling procedures were approved by the Rutgers University and New York University Animal Care and Facilities Committees.

#### Probes and surgery

Every animal was implanted with a four-shank diode probe as previously described (Stark et al., 2012). Probes were constructed by coupling 470 nm blue LEDs (diameter, 2 mm; model LB P4SG, Osram) to 50 μm multimode optical fibers and attaching every diode–fiber assembly to a single shank of a 32-site/four-shank silicon probe (Buzsaki32, NeuroNexus). Fiber tips were located ∼50 μm above the top recording site. Probes were implanted in the right hemisphere (anteroposterior, −1.6 mm; mediolateral, 1.1 mm) under isoflurane anesthesia. During surgery, the probes were lowered to a depth of 0.4–0.7 mm.

#### Recordings and photostimulation

After allowing the animals to recover for at least 48 h, recordings were initiated. Recordings were conducted in the home cage during spontaneous behavior. Mice were tethered by one ultralight cable for multichannel neuronal recordings and a second cable for multichannel optical stimulation. Recordings were conducted as the probe was moved gradually from the neocortex to the CA1 pyramidal cell layer. At each location in the brain, neuronal activity was inspected for spontaneous spiking activity, and, if encountered, a baseline period of at least 15 min was recorded followed by photostimulation (peak driving current, 60 mA; mean ± SD peak light power, 35 ± 7 μW; 50–70 ms pulses). Signals were generated by custom code written in MATLAB (MathWorks), converted by a digital signal processor (model RX5 and/or RX6, TDT) to voltage signals, and fed into a linear 16-channel current source. After each session, the probe was either left in place or moved (35–70 μm steps), and the brain was allowed to settle overnight.

#### Spike sorting and ground truth labels

During recordings, neural activity was filtered (1–5000 Hz), amplified (20× by Plexon headstages; 50× by an RC Electronics system), and digitized (16 bits, 20 kHz) on a 128-channel DataMax recording system (RC Electronics). Applied currents were recorded by the DataMax system. Offline, spike waveforms (32 samples/channel) were extracted from the wide-band records, detrended, and sorted into single units automatically (Harris et al., 2000), followed by manual adjustment. Only well isolated units [(amplitude, >50 μV; L-ratio, <0.05 (Schmitzer-Torbert et al., 2005); inter-spike interval index, <0.2 (Fee et al., 1996)] were considered. A total of 199 neocortical and 781 CA1 units conformed to these criteria.

**Figure 1.**
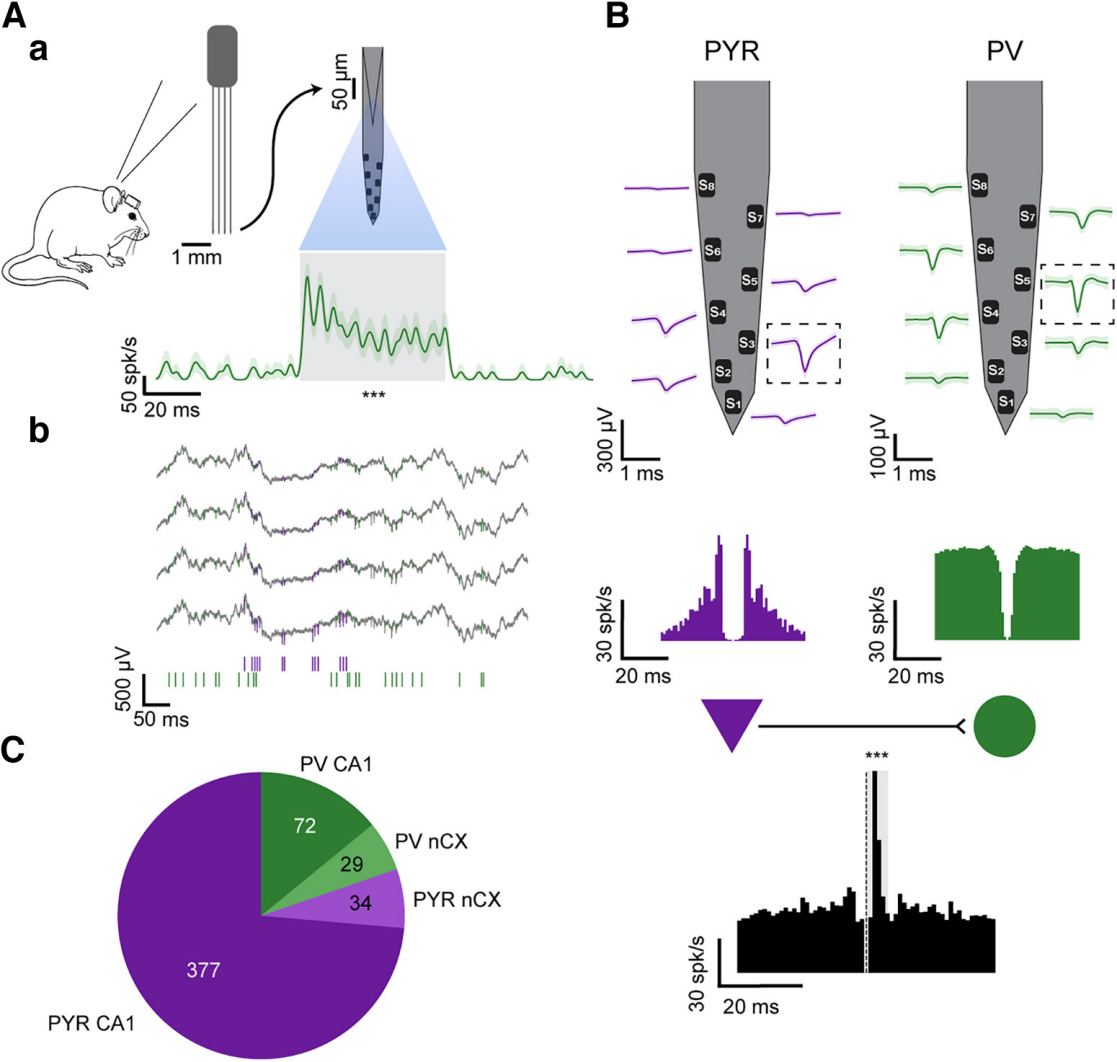
PYR and PV interneurons are tagged in freely moving mice. ***A***, Optical tagging of PV cells. ***a***, Every PV::ChR2 mouse was chronically implanted with a four-fiber/four-shank/32-channel optoelectronic array in the neocortex (nCX). Optical stimuli were applied, in separate sessions, in the nCX and in hippocampal region CA1. Peristimulus time histogram of the PV cell (bottom), triggered by the onset of 50 ms light pulses applied on the optical fiber attached to the recording shank (*n* = 20; 33 μW). The unit is tagged as PV because of a robust firing rate increase during light (gray) compared with no-light periods. ***p <* *0.001, Poisson test. ***b***, Wide-band (1–5000 Hz) recordings from four same-shank channels in CA1. Bottom, Spike trains of a PYR (purple) and the PV cell (green). ***B***, Connectivity-based tagging. Top, Mean (±SD) spike waveforms. For every unit, the channel that exhibits the highest trough-to-peak magnitude is denoted the main channel (boxed). Middle, Auto-correlation histograms (ACHs), showing burst spiking activity of the PYR (purple). Bottom, Cross-correlation histogram (CCH; black) between the spikes of the PYR and the optically tagged PV cell. Gray, Monosynaptic window. The CCH is consistent with monosynaptic excitation of the PV cell by the reference unit, tagging the reference unit as excitatory (PYR). ***p <* *0.001, Poisson test. ***C***, Tagged dataset. Of the 512 units in the dataset, 411 (80.3%) are PYR, and 449 (87.7%) are from CA1.

For connectivity-based tagging, we tagged units that participated as a reference in a cross-correlation histogram (CCH) that exhibited a significant (*p *<* *0.001, Bonferroni-corrected Poisson test) peak in the monosynaptic time range (0–5 ms) as excitatory cells (424 of 980 units). Units that exhibited a significant trough in the monosynaptic time range were tagged as inhibitory (21 of 980 units). For optogenetic-based tagging, units that exhibited a significant (*p *<* *0.001, Poisson test) increase in spiking rate during 50–70 ms DC pulses given on the recording shank were tagged as optically activated cells; 98 of 980 (10%) units conformed to the criterion. Next, we labeled units based on the three tags. Units tagged exclusively as excitatory were labeled as PYR cells (420 units), whereas units that were optically activated or inhibitory (but not excitatory) were labeled as PV cells (102 units). The remaining units (458 of 980, 47%) were not labeled and were discarded from the dataset. Ten of the labeled units (PYR 9 units; PV, 1 unit) were recorded using seven instead of eight channels and were therefore discarded as well, yielding a final dataset that included 512 tagged units (PYR cells, 411 units; PV cells, 101 units; Fig. 1). Of the 101 units referred to as “PV cells,” 93 were optically activated (92%), 13 were both optically activated and inhibitory, and 8 units were only inhibitory. Spike width, firing rate, and bursting behavior were similar for the inhibitory and the optically tagged PV cells. Thus, the 8 inhibitory-tagged PV-like cells were grouped with the 93 optically activated cells, and the entire group was denoted as PV cells. A majority of the units (449 of 512 units) were recorded from CA1. The median [interquartile range (IQR)] number of spikes per unit was 8368 [4494–16,929] for PYR, and 66,850 [13,031–174,802] for PV cells.

### Classification

#### Feature extraction

The shape and timing of the recorded spikes were used to extract a total of 34 features. We derived features of the following three modalities: waveform-based features, derived from a single channel (*n* = 8); spike-timing features, ignoring the spike waveform (*n* = 8); and spatial features, derived from the multichannel waveforms (*n* = 18). All features were based exclusively on spontaneous events that occurred in the lack of any light stimuli. For every spike, the waveform was extracted for 32 samples (1.6 ms) on every channel of the recording shank. The limited duration of the spikes places an upper bound on the classification performance of waveform-based and spatial models. For every channel separately, the waveform was averaged over spikes, and the mean waveform was calculated and upsampled by eightfold using Fourier interpolation to increase the temporal resolution. Since the average and the Fourier transform are linear operators, the order of the two steps does not affect the outcome.

#### Single-channel feature extraction

**Figure 2.**
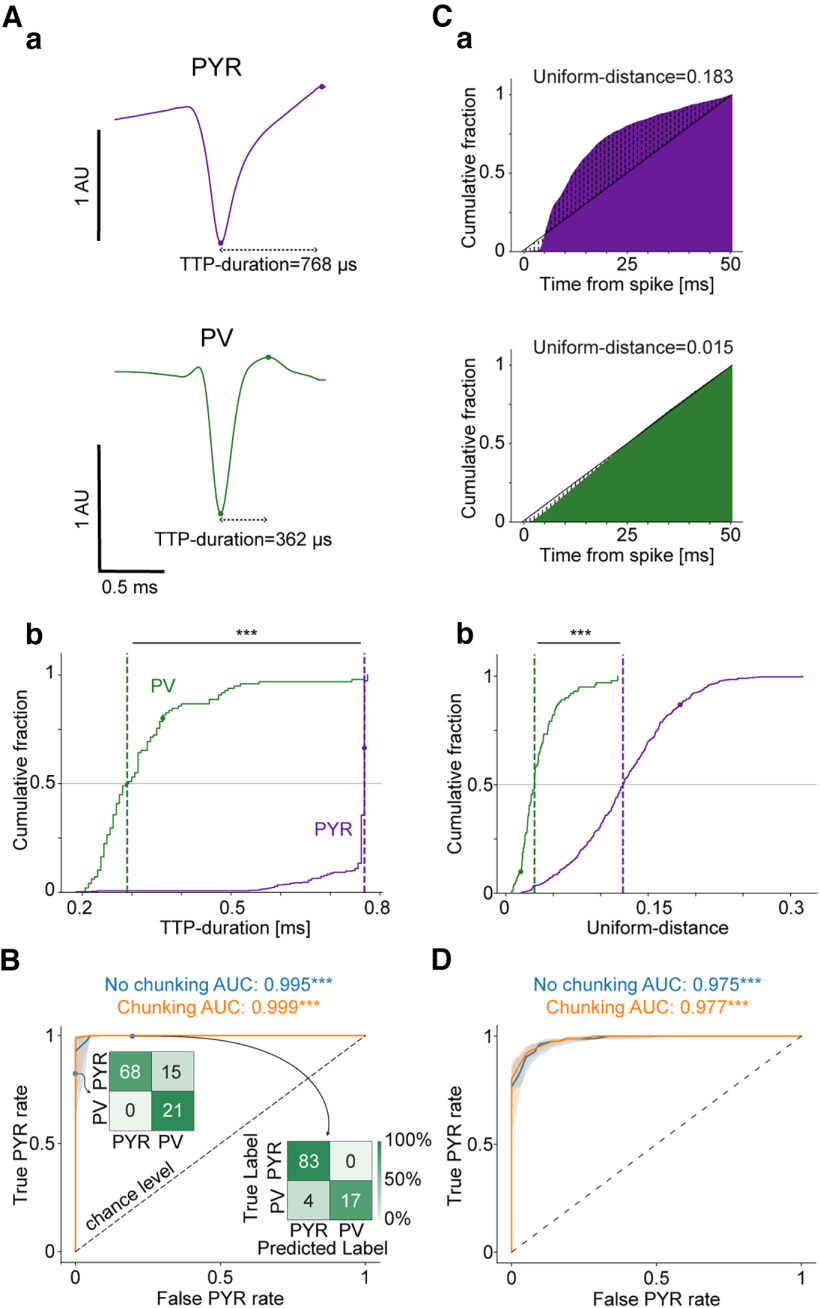
Waveform-based and spike-timing features allow near-perfect classification of PYR and PV cells. ***A***, Features extracted from the mean waveform of the main channel. Voltage values were scaled by the absolute value at the maximal negativity, yielding arbitrary units (AU; the example units are the same as in Fig. 1*B*). ***a***, Trough-to-peak (TTP) duration feature, defined as the time between the maximal negativity and the ensuing maximal positivity. ***b***, Cumulative distribution function (CDF) of the TTP duration feature for the entire population (PYR, *n* = 411; PV cells, *n* = 101; no chunking). Here and in all subsequent CDFs, horizontal lines represent 50%, vertical dashed lines indicate medians, and the *** symbol corresponds to *p *<* *0.001 (*U* test). The filled circles represent values corresponding to the examples given in ***a***. The difference between PYR and PV cells indicates longer TTP durations for PYR compared with PV cells. ***B***, Waveform-based features allow near-perfect classification. Cross-validated random forest models were trained using the waveform-based features (*n* = 50 partitions). The chunking method yields improved classification compared with no chunking. The ROC AUCs without chunking (blue) and with 50 spike chunks (orange) are higher than chance level. ***p <* *0.001, Wilcoxon test compared with chance level, 0.5. Inset, Confusion matrices (no chunking) based on different decision thresholds (top, 0.1; bottom, 0.9) show variability in prediction, exemplifying the shortcomings of threshold-dependent metrics. ***C***, Features extracted from spike timing. High-frequency features derived from single-sided short-term (0–50 ms) ACHs. ***a***, The Uniform-distance feature is defined as the average absolute difference between the single-sided ACH and a straight line (the example units are the same as in Fig. 1*B*). ***b***, Cumulative distribution of the Uniform-distance feature for the entire population (no chunking). The larger Uniform-distance values for PYR indicate larger deviations from linear recovery for PYR compared with PV cells. ***D***, Classification based on spike-timing features is not consistently improved by chunking. AUCs were derived from ROC curves based on *n* = 50 cross-validated random forest models. ROC curves for the test data without chunking (blue) and with 1600 spike chunks (orange) with performance above chance level. All conventions are the same as in ***B***. See also Extended Data Figures 2-1, 2-2.

For the waveform-based features, the channel with the largest trough-to-peak (TTP) magnitude was denoted as the “main” channel. The waveform in the main channel was scaled by dividing all values by the minimal value (i.e., at the trough). Scaling was performed to remove information about the sampling process (e.g., electrode impedance and neuron–electrode distance). When the absolute value at the trough was smaller than at the peak, waveforms were inverted (multiplied by −1). The outcome is a 256-element vector limited to the −1 to 1 range, with at least one value at −1. To provide a rich description of the waveform, a total of *n* = 8 waveform-based features were extracted from the main channel (Fig. 2, Table 1): four from the waveform itself, one from the first temporal derivative, and three from the second temporal derivative. For every feature, we compared the distribution of values between all available PYR cells (*n* = 411) and PV cells (*n* = 101) and calculated effect sizes. The specific measure of effect size used was the nonparametric estimator for common-language effect size (A_w_; Ruscio, 2008) which exhibits a smaller bias compared with alternatives (Li, 2016). A_w_ estimates the probability that a random sample from one distribution is larger than a random sample from a second distribution. Disregarding the direction of the effect, A_w_ is thus limited to the 0.5–1 range, taking a value of 0.5 when the two distributions are fully intermixed, and 1 when the two distributions do not overlap at all. All eight features (100%) exhibited a consistent difference (0.64 ≤ A_w_ ≤ 0.98; *p *< 0.05, *U* test; Table 1). Thus, all waveform-based features are potentially useful for classification.

**Table 1:** Waveform-based features

Feature	Family	Description	PYR median [IQR]	PV median [IQR]	Effect size (A_w_)^*a*^	Cell type (*p*-value)^*b*^	SHAP (*p*-value)^*c*^
TTP duration	Waveform	The duration between the trough (maximal negativity) and the peak (maximal positivity) [ms)	0.77 [0.76–0.77]	0.29 [0.25–0.35]	0.98	5.9 × 10^−58^	0.25 (0.001)
TTP magnitude		The difference between the trough and the peak (AU)^*d*^	1.4 [1.4–1.5]	1.3 [1.2–1.3]	0.97	3.7 × 10^−48^	0.11 (0.003)
FWHM		The duration in which the value is at least −0.5 (i.e., half of the trough) (ms)	0.21 [0.2–0.23]	0.16 [0.15–0.18]	0.89	3.7 × 10^−34^	0.005 (0.59)
Rise coefficient		A straight line connects the trough and the last sample. The coefficient is the time from the trough to the point where the absolute distance from the line is maximal (ms)	0.29 [0.26–0.32]	0.24 [0.21–0.26]	0.80	2.2 × 10^−21^	0.005 (0.99)
Maximum speed	First time derivative^*e*^	The duration after the trough, for which the spike maintains the same change rate (derivative) (ms)	0.19 [0.14–0.26]	0.14 [0.13–0.17]	0.69	3.4 × 10^−9^	0.011 (0.41)
Break measure	Second time derivative^*e*^	The sum of the values of the second derivative just before the trough (0.3-0.08 ms before the trough) (10–1 *AU)	−0.67 [−0.76 to −0.56]	−0.58 [−0.7 to −0.49]	0.64	7.9 × 10^−6^	0.003 (0.84)
Smile-cry		The sum of the values of the second derivative at the end of the spike (0.26-0.76 ms from the trough) (10–2 *AU)	−1.2 [−1.4 to −1.1]	−0.3 [−0.9 to −0.1]	0.89	5.7 × 10^−35^	0.013 (0.044)
Acceleration		The sum of the squared values of the second derivative just after the trough (0.08-0.25 ms after the trough) (10–6 *AU)^*b*^	9 [5–14]	91 [49–139]	0.97	6.8 × 10^−49^	0.12 (0.002)

a A_w_ ranges from 0.5 (no difference) to 1 (nonoverlapping distributions).

b Mann–Whitney *U* test.

c Median SHAP values based on 50 spike chunks, indicating feature importance. In parentheses are *p*-values based on a one-tailed permutation test.

d The waveforms are scaled to the −1 to 1 range. Thus, while the original units are μV, here we use arbitrary units (AU).

e Derivatives were computed numerically as the difference between every two adjacent samples.

The addition of a redundant feature would contribute no additional information to the classification process. To estimate relations between features, we first computed rank (Spearman’s) correlation coefficients (CCs) between every possible pair of waveform-based features (Extended Data Fig. 2-1*A*). To go beyond monotonic relations, we estimated mutual information (MI) between distributions of pairs of features (Timme and Lapish, 2018). If a feature had <10 unique values, the feature was considered naturally discrete; only the spatial dispersion (SPD)-Count feature (see Spatial feature extraction subsection below) was naturally discrete. The use of 10 bins limits the maximal information to log_2_10 = 3.3 bits. Deviation from chance level was determined using a permutation test, creating a null distribution based on 5000 iterations of shuffled pairs and evaluating the tail of the null distribution above the observed MI. We found that some features were only weakly correlated with others (e.g., the Break-measure, median [IQR] absolute CC was 0.19 [0.076–0.24]), suggesting that independent information could be gleaned by using the feature. Alternatively, a weakly correlated feature may be dominated by noise. However, the possibility is unlikely since the Break-measure differed for the PYR and PV groups (A_w_ = 0.64; *p *=* *7.85 × 10^−6^, *U* test; Table 1). Other features were more strongly correlated with the host of other features (e.g., full-width at half-maximum (FWHM), 0.81 [0.45–0.87]; Smile-cry, 0.76 [0.33–0.91]). Since a small number of samples limits the power of standard statistical tests (e.g., the Mann–Whitney *U* test), when comparing CCs between two groups within a modality we applied a permutation test. We compared a statistic, defined as the difference between the medians of the two groups of CCs, to the 95th percentile of a null distribution created by shuffling the CCs between the groups, and calculating the statistic 1000 times. We did not observe stronger absolute CCs within families: the absolute intrafamily correlation was 0.37 [0.14–0.62], whereas the absolute interfamily CC was 0.47 [0.24–0.79] (*p *>* *0.05, permutation test). Quantifying the interrelations between waveform-based features using MI yielded similar results. The median [IQR] MI between waveform-based feature distributions was 0.658 bits [0.292–0.955] bits, and the rank correlation coefficient between the MI and CCs was 0.816 (*p *=* *0.001, permutation test; Extended Data Fig. 2-1*B*, inset). The bulk of the variance in the MI (*R*^2^ = 0.67) could be explained by pairwise rank correlations, suggesting that interrelations between feature pairs are largely monotonic. Thus, based on the feature redundancy analysis, partitioning into families may have only semantic value, and all derived features may contribute to classification.

10.1523/ENEURO.0265-22.2022.f2-1 Figure 2-1 Extended data for Figure 2. Waveform-based feature interrelations. ***A***, Rank (Spearman’s) correlations between waveform-based features, grouped by families. Most correlations (26 of 28; 93%) differ from zero. Blank, *p *>* *0.05; *p <* *0.05; **p <* *0.01; ***p <* *0.001; permutation test. ***B***, MI between waveform-based features. All pairs (28 of 28; 100%) exhibit MI values that are higher than chance level. ***p <* *0.001, permutation test. Inset, Scatter plot of the MI values between pairs of features and the pairwise absolute rank CCs from ***A*** with statistics for rank (Spearman’s) correlation. Download Figure 2-1, TIF file.

#### Spike-timing features

For the features based on spike timing, the autocorrelation histogram (ACH) was calculated for every unit over a range of ±1000 ms using a bin size of 0.5 ms. The ACH depends only on the timing of the spikes and is agnostic to the spike waveforms. The ACH was upsampled eightfold using polyphase filtering to increase the temporal resolution. The ACH is an even function, but edge effects necessarily cause asymmetry in any practical implementation. To obtain an ACH (0–1000 ms) free from edge effects, the value in every positive time bin was averaged with its negative homolog. A total of *n* = 8 spike-timing features was derived (Fig. 2, Table 2). Three of the features were high-frequency features, derived from the short-term ACH (up to 50 ms). Two were low-frequency features, derived from the long-term ACH (50–1000 ms). The last three were wide-band features: two were derived from the complete ACH (0-1000 ms), and one was derived from the entire recording. For every feature, we compared the values derived for the PYR and PV cells. All eight features (100%) exhibited a consistent difference between PYR and PV cells (*p *<* *0.05, *U* test; Table 2). Thus, spike-timing features may be useful for classification.

**Table 2:** Spike-timing features

Feature	Family	Description	PYR median [IQR]	PV median [IQR]	Effect size (A_w_)^*a*^	Cell type *p*-value^*b*^	SHAP (*p*-value)^*c*^
Uniform- distance	High frequency (0–50 ms)^*d*^	The mean distance between the CDF of the ACH and the CDF of a uniform distribution	0.12 [0.09–0.16]	0.03 [0.02–0.04]	0.95	1.6 × 10^−44^	0.14 (0.002)
D_KL_-Short		The D_KL_ between the PDF of the ACH and the PDF of a uniform distribution	0.25 [0.17–0.34]	0.058 [0.037–0.079]	0.95	2.2 × 10^−44^	0.025 (0.36)
Rise time		The duration in which the values in the CDF of the ACH are above a threshold of 1/e (ms)	11.1 [9.4–13.7]	19.3 [17.8–20.6]	0.88	4.3 × 10^−33^	0.031 (0.25)
Jump-index	Low frequency (50-1000 ms)^*d*^	The mean distance between the CDF of the ACH and the CDF of a uniform distribution	0.067 [0.044–0.088]	0.016 [0.0095–0.024]	0.92	9.3 × 10^−39^	0.029 (0.31)
D_KL_-Long		The D_KL_ between the PDF of the ACH and the PDF of a uniform distribution	0.069 [0.032–0.15]	0.0039 [0.0017–0.012]	0.89	6.1 × 10^−35^	0.19 (0.001)
PSD-center	Wide-band (0–1000 ms)	The centroid of the power spectral density (PSD), namely the squared FFT of the ACH (Hz)	37 [33–42]	31 [26–37]	0.65	2.8 × 10^−6^	0.016 (0.56)
PSD′-center		The centroid of the derivative^*e*^ of the PSD with respect to frequency (Hz)	23 [19–29]	21 [18–27]	0.57	0.012	0.009 (0.83)
Firing rate		The average firing rate (spikes/s)	0.69 [0.35–1.47]	8.95 [3.39–16.35]	0.93	1 × 10^−40^	0.077 (0.038)

a A_w_ ranges from 0.5 (no difference) to 1 (nonoverlapping distributions).

b Mann-Whitney *U* test.

c Median SHAP values based on 1600 spike chunks, indicating feature importance. In parentheses are *p*-values based on a one-tailed permutation test.

d Most high-frequency and low-frequency features are based on distributions and therefore hold no units.

e Derivatives were computed numerically as the difference between every two adjacent samples.

To quantify interrelations, we computed CCs and MI between spike-timing features extracted from the complete spike trains (Extended Data Fig. 2-2). The high-frequency features exhibited high within-family absolute correlations (median [IQR], 0.85 [0.83–0.88]), whereas lower absolute correlation values were observed between the other features (low-frequency and wide-band families together: 0.26 [0.24–0.70]; *p *=* *0.098, permutation test; Extended Data Fig. 2-2*A*). In contrast to the waveform-based feature families, intrafamily absolute correlations (0.81 [0.49–0.83]) were higher than interfamily absolute correlations (0.50 [0.40–0.61]; *p *=* *0.012, permutation test). MI between pairs of spike-timing features yielded results similar to those for pairwise correlations. The median [IQR] MI between spike-timing features was 0.469 [0.283–0.701] bits, and the rank correlation coefficient between MI and CCs was 0.967 (*p *=* *0.001, permutation test; *R*^2^ = 0.93; Extended Data Fig. 2-2*B*, inset). The fact that almost all variance of the MI values is explained by pairwise correlations suggests that the interrelations between the pairs of features are largely monotonic. The correlations between high-frequency features, namely Uniform-distance, Kullback–Leibler distance (D_KL_)-Short, and Rise-time suggest that the features provide redundant information. Thus, a small number of spike-timing features may suffice for classification.

10.1523/ENEURO.0265-22.2022.f2-2 Figure 2-2 Extended Data for Figure 2. Spike-timing feature interrelations. ***A***, Rank correlations between the spike-timing features grouped by families. Most correlations (27 of 28; 96%) differ from zero. All conventions here and in ***B*** are the same as in Extended Data Figure 2-1. ***B***, MI between spike-timing features. All pairs (28 of 28; 100%) exhibit MI values that are higher than chance level. Inset, Scatter plot of the MI between pairs of features and the CCs from ***A***. Download Figure 2-2, TIF file.

#### Spatial feature extraction

**Figure 3.**
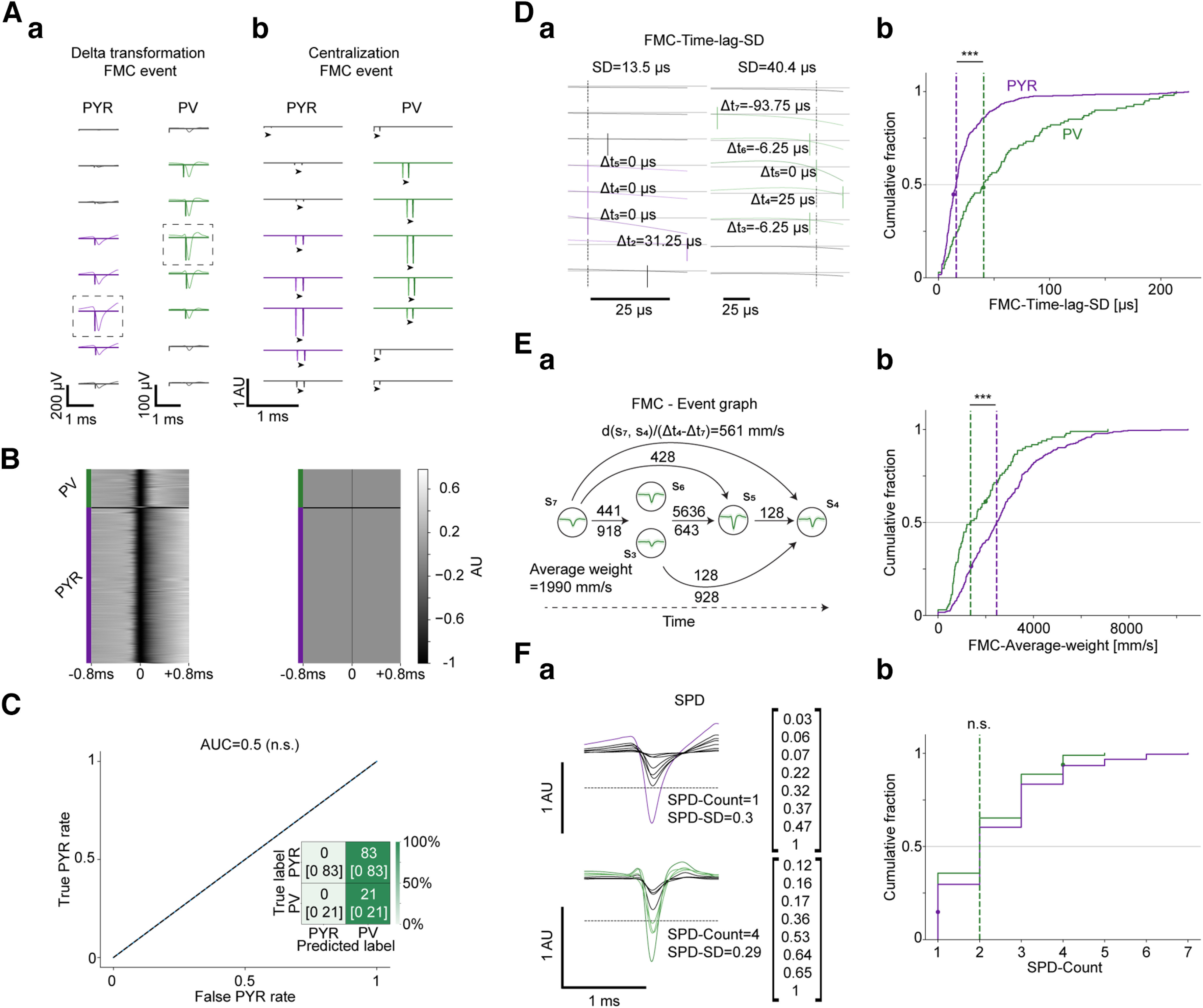
Transforming multichannel spike waveforms to event-based δ-like functions removes all waveform-based information and allows extracting purely spatial features. ***A***, The event-based δ-transformation procedure, illustrated for the FMC event. ***a***, The mean waveforms, with δ-like functions marking the FMCs. The transformation replaces all voltage values with zeros, except for the event points, which are assigned the same value as the trough. In gray are channels for which the magnitude of the TTP is below a predetermined threshold (Materials and Methods). The main channels are boxed. ***b***, Next, waveform-related information that might be recovered by combining multiple δ-transformed events is removed. The δ-like functions are scaled and centralized (arrowheads), placing the event of the main channel at the midpoint (129th sample). ***B***, Left, The scaled waveform of the main channel of all units in the dataset before the transformation, sorted for PYR and PV cells separately by the time of the trough. Right, The same waveforms after event-based δ-transformation. The transformation removes nearly all of the variability between units. ***C***, Cross-validated random forest models (*n* = 50; no chunking) were trained using waveform-based features extracted from the transformed spikes. The confusion matrix, based on a naive decision threshold of 0.5, yields a constant prediction of one class. n.s.*p *>* *0.05, Wilcoxon test. Numbers in every cell denote the median [IQR]. Performance was quantified by the threshold-independent AUC. The classification yields an AUC of exactly 0.5, corresponding to purely random prediction. ***D***, A time-based feature, FMC-Time-lag-SD, derived from the differences between the times of the FMC event in different channels. The feature quantifies the temporal dispersion of the event, without considering the actual positions of the recording electrodes. ***a***, FMC-Time-lag-SD is defined as the SD of the time differences between the FMC event of the main channel (vertical dotted lines) and the other channels. In gray are ignored channels, for which the magnitude of the TTP was below a predetermined threshold. ***b***, Cumulative distribution of the FMC-Time-lag-SD feature for the entire population (411 PYR, 98 PV cells, no chunking). The smaller FMC-Time-lag-SD values of the PYR indicate higher spatiotemporal synchrony for PYR compared with PV cells. All conventions for the CDFs here and in subsequent panels are the same as in Figure 2*A*. ***E***, A graph-based feature, FMC-Average-weight, derived from the differences between the FMC event time in different channels and the electrode locations. ***a***, FMC-Average-weight is defined as the average edge weight in the event graph. The event graph is a directed graph with vertices representing the electrodes, and edges representing the transmission speed based on the timing of the events and the location of the electrodes. Only channels that passed the threshold for the magnitude of the TTP were considered. ***b***, Cumulative distribution of the FMC-Average-weight feature (no chunking). The larger values for PYR indicate higher transmission rates for PYR compared with PV cells. ***F***, A value-based feature, SPD-Count, derived from SPD of the maximal negativity on every channel. ***a***, SPD-Count is defined as the number of channels that reached at least 50% of the maximal negativity of the main channel. ***b***, Cumulative distribution of the SPD-Count feature (no chunking). No consistent difference between the PYR and PV cells is observed, suggesting similar spatial distributions of the scaled maximal negativity (*p *>* *0.05, *U* test). See also Extended Data Figure 3-1.

For extracting purely spatial features, an event-based δ-transformation was first applied to the mean upsampled waveform of every channel to remove all waveform-based information (Fig. 3*A*), as follows. (1) First, positive spikes were inverted as done for the waveform-based process. (2) Next, three events were defined. One event was the time of maximal negativity (NEG). For the additional two events, the median over all the channels was calculated. The second event was the first median crossing (FMC), the first time point before the maximal negativity of the channel in which the global median was crossed. The third event was the second median crossing (SMC), the first time point after the maximal negativity of the channel in which the global median was crossed. Every event was detected on every channel, yielding a total of 24 points. (3) Third, the waveform was replaced by a δ-like function that took the value of the maximal negativity of the channel at the singular event time point and zeros everywhere else. The δ-like functions were scaled by the absolute value of the global minimum over all channels, effectively removing all waveform-based information from every single channel. However, waveform-based information may still be available when using multiple events together (e.g., FMC, NEG, and SMC). (4) To remove all waveform-based information, the δ functions were shifted together to centralize (shift to the 129th sample) the time of the event on the main channel. The process transforms the waveform in the main channel to be nearly identical for all units (Fig. 3*B*). Residual variability in the time of the trough may remain if the channel with maximal magnitude of the TTP and the channel with the maximal negativity are not the same.

10.1523/ENEURO.0265-22.2022.f3-1 Figure 3-1 Extended Data for Figure 3. Spatial feature interrelations. ***A***, Correlations between the spatial features, grouped by families. Eighty percent of the feature pairs (122 of 153) exhibit correlations that differ from zero. All conventions here and in ***B*** are the same as in Extended Data Figure 2-1. ***B***, MI between spatial features. Most pairs (126 of 153; 82%) exhibit MI values that are higher than chance level. Inset, Scatter plot of the MI and the absolute CCs from ***A***. Download Figure 3-1, TIF file.

Overall, *n* = 18 features were extracted from the transformed waveforms (Table 3) associated with the three events, quantifying the following three dimensions: time based, graph based, and value based. (1) In the time-based dimension, only the timing of the events was considered (e.g., the SD of the FMC; Fig. 3*D*, FMC-Time-lag-SD). Channels for which the magnitude of the TTP (before δ-transformation) did not pass an arbitrary threshold of 25% of the maximal magnitude of the TTP over all channels were ignored (Fig. 3*D*, gray). A median [IQR] of 4 [3–5] PYR channels and 3 [2–5] PV cell channels were removed. (2) The graph-based dimension included both event timing and the physical locations of the recording electrodes on the probe. An event graph was generated based on a specific event, with a node for each channel (e.g., Fig. 3*E*, FMC-Average-weight). Only channels for which the magnitude of the TTP passed the 25% threshold were considered. Directed edges connected every two nonoverlapping events, with a weight representing “transmission speed”: the Euclidean distance between the electrodes, divided by the time difference between the events. (3) The value-based dimension (SPD) ignored timing information and considered the scaled maximal negativity values on every channel, based on the global maximal negativity (e.g., Fig. 3*F*, SPD-Count). We found that 10 of 18 (56%) of the spatial features exhibited differences between PYR and PV cells (*p *<* *0.05, *U* test). Although some features do not show consistent differences between the two cell types, classification may benefit from the features because of, for instance, distinct second-order statistics.

**Table 3:** Spatial features

Feature	Family	Description	Event	PYR median [IQR]	PV median [IQR]	Effect size (A_w_)^*a*^	Cell type *p*-value^*b*^	SHAP (*p*-value)^*c*^
Time-lag-SS	Time based^*d*^	The mean SS of the time offsets of the event (103 * μs2)	FMC	0.44 [0.13–1.39]	3.33 [0.49–12.02]	0.74	6.47 × 10^−14^	0.094 (0.001)
			NEG	0.34 [0.1–1.1]	0.16 [0.05–0.35]	0.64	7.04 × 10^−6^	0.008 (0.32)
			SMC	1.9 [0.77–3.45]	1.69 [0.68–3.48]	0.51	0.34	0.046 (0.015)
Time-lag-SD		The SD of the time offsets of the event (μs)	FMC	15.9 [8.8–26.3]	40.6 [17.4–86.3]	0.72	2.3 × 10^−12^	0.093 (0.001)
			NEG	13.5 [7.7–23.5]	9.4 [5.4–13.3]	0.63	3.44 × 10^−5^	0.009 (0.30)
			SMC	26 [18.8–34.3]	24.1 [15.6–38.9]	0.50	0.47	0.052 (0.006)
Average-weight	Graph-based	The average edge weight in the graph (mm/s)	FMC	2455 [1366–3509]	1206 [684–2577]	0.68	1.65 × 10^−8^	0.038 (0.027)
			NEG	2724 [1678–4269]	3633 [2173–5387]	0.61	3.13 × 10^−4^	0.007 (0.31)
			SMC	1844 [1232–2674]	2034 [1099–2947]	0.51	0.34	0.008 (0.31)
Longest path		The sum of weights in the longest path in the graph (mm/s)	FMC	6722 [3581–11,514]	4909 [2417–9945]	0.58	0.0053	0.014 (0.18)
			NEG	8103 [4200–14,436]	11,517 [4923–16,349]	0.55	0.06	0.006 (0.33)
			SMC	6050 [3269–11,115]	6888 [3237–11,056]	0.51	0.33	0.013 (0.21)
Shortest path		The sum of weights in the shortest path in the graph (mm/s)	FMC	1049 [657–1619]	494 [267–928]	0.74	1.04 × 10^−13^	0.068 (0.001)
			NEG	1071 [714–1754]	1607 [916–2572]	0.63	1.4 × 10^−5^	0.006 (0.33)
			SMC	584 [426–872]	701 [409–1024]	0.55	0.08	0.008 (0.30)
SPD- Count	Value-based^*e*^	The number of values that crossed 0.5		2 [1–3]	2 [1–3]	0.55	0.06	0.007 (0.17)
SPD-SD		The SD of the vector		0.30 [0.28–0.33]	0.29 [0.27–0.31]	0.65	3.02 × 10^−6^	0.008 (0.32)
SPD-Area		The AUC^*f*^		1.95 [1.45–2.54]	2.02 [1.64–2.44]	0.52	0.29	0.016 (0.19)

SS, Sum of squares.

a A_w_ ranges from 0.5 (no difference) to 1 (nonoverlapping distributions).

b Mann-Whitney *U* test.

c Median Shapley additive explanations (SHAP) values based on 25 spike chunks, indicating feature importance. Parentheses, *p*-values based on a one-tailed permutation test.

d Time offsets are relative to the main channel.

e Based on the vector of maximal negativity values for each channel, scaled to the 0–1 range. The features are based on counts and thus hold no units.

f The area under the curve of the count of channels versus the threshold value.

To estimate feature redundancy because of high correlations, we computed the CCs between the spatial features extracted from the transformed mean waveforms (Extended Data Fig. 3-1*A*). The rank correlation matrix of the spatial features showed absolute correlations (median [IQR]: 0.2 [0.1–0.33]), that were weaker than for the waveform-based features (*p *=* *3.5 × 10^−4^, *U* test) and for the spike-timing features (*p *=* *1.1 × 10^−7^). Eighty percent of the spatial feature pairs exhibited absolute correlations higher than zero (122 of 153; *p *<* *0.05 permutation test). Intrafamily absolute correlations (0.27 [0.17–0.42]) were higher than interfamily correlations (0.17 [0.08–0.29]; *p *= 0.006, permutation test). The median [IQR] MI between spatially based feature distributions was 0.19 [0.155–0.266] bits, and the rank correlation coefficient between MI and CCs was 0.84 (*p *=* *0.001, permutation test; *R*^2^ = 0.706; Extended Data Fig. 3-1*B*). Since the correlation and the MI analysis agreed, the relatively weak correlations between spatial features may result from large amounts of noise in every feature. Alternatively, the features may provide independent information, useful for classification.

#### Classification procedure

The classification model was chosen to be random forests (Breiman, 1996, 2001) because of the relative simplicity. Furthermore, several methods are available for understanding the determinants of a specific random forest model prediction (Archer and Kimes, 2008). To achieve good estimation of model performance, a nested cross-validation procedure was applied (Varma and Simon, 2006; Krstajic et al., 2014). For every modality (waveform, spike timing, and spatial), the training procedure was repeated *n* = 50 times. In every iteration, data were first partitioned in an approximate 80:20 ratio into training and test sets in a stratified fashion. Thus, the training set always included 328 PYR and 80 PV cells, and the test set included 83 PYR and 21 PV cells; only the identity of the units changed between iterations. To handle the imbalance between the number of PYR and PV cells in the dataset, the model weights that control the effect of every class on the impurity score used for training the random forest model were adjusted. Specifically, instead of assigning equal weights, class weights were set to be inversely proportional to the number of samples in every class using the following: total number of training set samples/(number of classes × number of class samples in the training set). Second, a fivefold grid search was conducted on the training set to find the best hyperparameters for the model, optimizing the receiver operating characteristic (ROC) of the area under the curve (AUC). The tested hyperparameters were the number of estimators, the depth of each estimator, the minimal number of samples required to split a node, and the minimal number of samples required to be at a leaf node. Other hyperparameters received default values based on the implementation of the *scikit-learn* library in Python (Pedregosa et al., 2011). Third, using the optimized hyperparameters, the model was trained on the entire training set. Finally, model performance was evaluated using the test set.

#### Performance and explainability

Model performance was assessed using a metric that is robust to unbalanced data. Two types of metrics exist: threshold dependent and threshold independent. Threshold-dependent metrics consider only the binary decision: in our case, PYR or PV. Threshold-independent metrics consider the raw prediction, a value between 0 and 1, and not the decision itself. Threshold-dependent metrics require choosing a threshold, and thus the outcome may vary according to the chosen decision threshold. An arbitrarily chosen threshold does not necessarily reflect performance, especially when considering unbalanced datasets (Sheng and Ling, 2006). The choice of a decision threshold is not trivial, and is the subject of active research (Esposito et al., 2021). For these reasons, we used an established threshold-independent metric, the AUC (Fawcett, 2006). The AUC reflects the relation between true positives and false positives for all possible thresholds and is hence threshold independent as well as suitable for unbalanced datasets.

The theoretical chance level for the AUC metric is 0.5. To determine the empirical chance level, the performance of models trained on data with shuffled training-set labels was assessed. For every modality (waveform, spike timing, and spatial), the training procedure was conducted with the labels shuffled only for the training set. The AUC exhibited the expected chance-level behavior, yielding the following median [IQR] values: waveform, 0.46 [0.34–0.57]; spike timing, 0.46 [0.39–0.56]; and spatial, 0.48 [0.44–0.55]. Since chance-level results were obtained on the test sets, further assessment of model performance was conducted with respect to 0.5, the theoretical chance level.

To assess the contribution of every feature to the final classification prediction, we used Shapley additive explanations (SHAPs) values (Lundberg et al., 2020). The SHAP method uses a game-theoretic approach to calculate an importance score for every feature in each sample, considering all interdependencies with other features. Since the predictions of random forest models range from 0 to 1, the contribution of every feature can take values from −1- to 1. For each model, we calculated and averaged the absolute contribution of every feature based on the test set, taking the median over the 50 partitions to avoid idiosyncrasies of a single arbitrary partition. Since the absolute value is taken, SHAP values are necessarily non-negative, creating a skewed distribution that does not have an expected value of zero. To calculate significance, we used a permutation test. The SHAP values for each feature were compared with the SHAP values obtained for the models trained with shuffled training-set labels. Models trained with shuffled labels represent chance-level classifiers, for which the importance of every feature can be considered as the chance-level baseline. To create a null distribution, we partitioned the dataset into a train and test sets 1000 times. For every train-test partition, we shuffled the labels, trained the models as described previously, and calculated the SHAP values. Then, to calculate a *p*-value for every feature, we compared the original SHAP value to the null distribution.

#### Chunking method

Models trained on larger nonredundant datasets typically exhibit improved performance. Therefore, data augmentation approaches to synthetically increase the size of the dataset are often applied (Moreno-Barea et al., 2018). Augmentation may be implemented by adding noise or transforming the data (rotation and reflection in image classification; Mikołajczyk and Grochowski, 2018). Here, to augment the size of a given dataset, features were extracted from “chunks” that included subsets of spikes, instead of using all available spikes together. Thus, we increased variability using the natural richness of the data that is otherwise flattened to a single mean waveform. The chunking process increases the number of samples in the dataset, with the possible cost of increased noise.

A total of *n* = 7 different chunk sizes was used, with 25, 50, 100, 200, 400, 800, or 1600 spikes per chunk. For a given unit with *N* spikes and a chunk size *C*, the spikes were randomly split into ⌊*N*/*C*⌋ chunks so that every chunk consisted of between *C* and 2*C* – 1 distinct spikes. Spikes were randomly assigned to chunks. In a given split, all chunks consisted of the same number of spikes up to a difference of a single spike. Every chunk received the label of the source unit. Because of the different numbers of spikes recorded for PYR and PV cells (5,651,196 PYR spikes and 11,612,978 PV cell spikes), the balance between PYR and PV samples changed compared with the original dataset (411 PYR and 101 PV cells). For instance, using a chunk size of 25 spikes, the total number of PYR samples was 225,850 while the number of PV samples was 464,473.

To determine features for every chunk separately, the waveforms were averaged over all available same-channel spikes within a chunk, from which waveform and spatial features were derived. To derive chunk-specific spike-timing features, the ACH was accumulated by summing over all single-spike ACHs. For every spike, the single-spike ACH was based on all spikes that occurred in the −1000 to 1000 ms time window around the reference spike. To be applicable to the chunking case, the single-spike firing rate was defined as the mean of the inverse interspike intervals before and after the spike.

To provide information about the distribution of the values over chunks, several statistics were extracted from the individual values of every chunk. Extracting statistics based on all the chunks of a unit allows considering intraunit variability as a feature. For every feature, the mean, SD, and the 25%, 50%, and 75% quantiles were extracted (referred to as “chunk statistics”). Note that extracting the mean out of all the individual feature values of the chunks is not the same as not using chunking. Without chunking, first all the waveforms are averaged, transformed, and then features are extracted. In contrast, when using the mean over chunks, averaging happens at the end of the process (averaging the waveforms within a chunk still happens at the beginning). If all steps are linear, the two processes yield the same results. However, since feature extraction is not a linear operator, the mean statistic may contain unique information. The specific statistics were chosen to capture the first and second moments of the across-chunk distribution and for their simplicity. The chunk statistics can be expanded further to capitalize on different properties of the distribution over different chunks. The five new chunk statistics increased the total number of features used by the model sixfold. Notably, the chunk statistic features are the same for all the chunks of a given unit.

When training models using chunks, the data were partitioned based on units. In the training set, every chunk was considered independently (not as part of the unit). In addition, instead of performing a grid search for every chunk size, the hyperparameters for all chunk sizes were chosen by a no-chunking equivalent. Chunk statistics were extracted for the no-chunking dataset as well, so the number of features was equal to the number of features in the chunking method. The models applying chunking used the hyperparameters found using the no-chunking equivalent grid search, based on the same partition of the data to training and test sets. For testing and evaluating, every chunk received an independent prediction. Then, predictions were pooled over all same-unit chunks by casting a majority vote, yielding a final chunk-based prediction for the unit.

When calculating the SHAP values for the chunking-based models, we randomly chose 1000 samples out of the test set. The procedure for computing SHAP values for chunking-based data were otherwise the same as for the no-chunking data. The absolute SHAP value of every feature was summed together with the values of the chunk statistics extracted from the same feature, yielding a single importance value for every original feature.

#### Generalization analysis

Units in the tagged dataset were recorded from CA1 (449 of 512 units, 88%) and from neocortex. The availability of tagged data from two brain regions allows testing and quantifying interregion generalization. Generalization was determined by the performance of models trained using recordings from one region on test data from the same region (“training region”), and from the other region (“non-trained-on region”). To directly quantify generalization, we partitioned the full dataset into the following three sets for each training region: (1) a training set, containing ∼80% of the training region units (CA1: 301 PYR, 58 PV; neocortex: 27 PYR, 23 PV); (2) a test set, containing the remaining 20% of the training region units (CA1: 76 PYR, 14 PV; neocortex: 7 PYR, 6 PV); and (3) a second test set, containing all units of the non-trained-on region (neocortex: 34 PYR, 29 PV; CA1: 377 PYR, 72 PV). Waveform, spike timing, and spatial models were trained on the reduced CA1 dataset with 50, 1600, and 25 spike chunks, respectively (found to yield the best performance for each modality on the combined dataset). Chunked data were used for the grid search: the training set was further partitioned into a “development set” containing 80% of the units and an “evaluation set” containing 20% of the units. If the development set contained >5000 chunks, the grid search was conducted on a random subset of 5000 chunks, minimizing run time while allowing an efficient search. The difference in the number of units between the CA1 and neocortical training sets leads to an inherent difference in absolute performance between the two training region conditions. However, generalization can be readily compared between the two conditions based on the performance of the test set of the non-trained-on region relative to the performance of the test set from the training region.

### Statistical analyses

A threshold of α = 0.05 was used for all statistical tests. An exception was the threshold used for tagging the units, namely for determining whether a unit exhibits light activation, and whether two units exhibit monosynaptic connectivity (α = 0.001). All descriptive statistics (*n*, median, IQR) can be found in the results, figures, tables, and legends. Differences between the medians of two unpaired groups were evaluated using a Mann–Whitney *U* test (two tailed unless otherwise specified). Differences between the median of a single group and a number, or between the medians of two paired groups, were evaluated using Wilcoxon’s signed-rank test (one tailed unless otherwise specified). Comparisons of more than two groups were conducted using a Kruskal–Wallis one-way nonparametric ANOVA, and corrected for multiple comparisons using Tukey’s procedure. Rank (Spearman’s) correlation coefficients were tested using a permutation test. All statistical tests were conducted using either *SciPy* library (Virtanen et al., 2020) or custom code implemented in Python and MATLAB.

### Data availability

The code used for feature extraction, model training, and visualization is freely available on GitHub (https://github.com/EranStarkLab/SpatiotemporalSpiking).

## Results

### PYR and PV interneurons are tagged in freely moving mice

Differentiating between PYR and PV cells based on electrical properties requires a ground truth-labeled dataset. We recorded and tagged extracellular spiking data from freely moving PV::ChR2 mice (*n* = 7) using chronically implanted four-shank, 32-channel optoelectronic arrays (Fig. 1*Aa*). Every shank was equipped with a diode-coupled fiber, enabling independent illumination of small local groups of neurons while concurrently recording the extracellular activity (Fig. 1*Ab*). We used 50–70 ms light pulses for optical tagging. A unit was tagged as PV if the stimulus-locked firing rate increase was consistently above baseline (*p *<* *0.001, Poisson test; Fig. 1*Ac*). Using the optical tagging procedure, a total of 27 units from the neocortex and 71 from CA1 were tagged as PV cells.

For every pair of simultaneously recorded units, we calculated the spike-to-spike CCH and tested for peaks in the monosynaptic time range (0–5 ms; *p *<* *0.001, Bonferroni-corrected Poisson test). Units that participated as a reference in a CCH that exhibited a significant peak were tagged as excitatory (Fig. 1*B*). Using the monosynaptic CCH analysis, 424 units were tagged as excitatory and 21 as inhibitory; 13 of 21 units were both inhibitory and optically activated. Together with the optically tagged PV cells and after removing invalid samples (Materials and Methods), the dataset consists of 512 units, of which 411 units (80.3%) are PYR (Fig. 1*C*).

### Waveform-based and spike-timing features allow near-perfect classification of PYR and PV cells

Waveform-based spike properties differ between PYR and PV cells and are widely used for cell type classification (Barthó et al., 2004; Cardin et al., 2009; Stark et al., 2013). However, many classifiers use waveform-based features in conjunction with features based on spike timing, and previously used spike-based classifiers have not been cross-validated. For every unit, we calculated *n* = 8 features based on the waveform of the main channel (e.g., TTP duration; Fig. 2*A*, Table 1), defined as the channel with the largest magnitude of the TTP. After deriving features for every unit, we trained and tested classification models. The median [IQR] AUC for the models was 0.995 ([0.978–1]; *p *=* *3.1 × 10^−10^, Wilcoxon test compared with chance level of 0.5). Despite the high AUC, model performance improved when spikes were partitioned into chunks of 25, 50, and 200 spikes (*p *<* *0.05, Wilcoxon test). Partitioning into 50 spike chunks increased the original AUC by 0.11% [0–0.55%] to 0.999 (0.989–1; *p *=* *0.001, Wilcoxon test; Fig. 2*B*). A feature importance (SHAP) analysis conducted on the models trained with 50 spike chunks (Table 1) indicated that the TTP-duration feature provided the largest contribution to the prediction (median [IQR] over all 50 instantiations: 0.25 [0.23–0.26], *p *=* *0.001, permutation test). The fact that the AUC is near unity means that models based strictly on waveform features achieve near-perfect classification.

While spike-timing information has been used for cell type classification before, most implementations also considered waveform-based features (Csicsvari et al., 1998; Frank et al., 2001; Viskontas et al., 2007). To directly test whether spike timing alone can yield accurate classification, we derived *n* = 8 spike-timing features from the spike trains of every unit (e.g., Uniform-distance; Fig. 2*C*, Table 2). We conducted the training and evaluation process for the spike-timing modality as for the waveform-based classification. Without chunking, the AUC was 0.975 [0.957–0.986] (*p *=* *3.8 × 10^−10^, Wilcoxon test). The performance of the spike-timing models did not exhibit consistent improvement on chunking (*p *>* *0.05 for all chunk sizes, Wilcoxon test). Nevertheless, the highest improvement in the AUC was achieved using 1600 spike chunks, increasing performance by 0.28% [−0.39 to 0.74%] to yield an AUC of 0.977 [0.965–0.987] (*p *=* *0.07, Wilcoxon test; Fig. 2*D*). SHAP analysis using models trained with 1600 spike chunks (Table 2) attributed the highest importance to the D_KL_-Long feature (0.19 [0.17–0.2]), followed by the Uniform-distance feature (0.14 [0.12–0.16] (*p *<* *0.002 for both, permutation test). The results suggest that both high-frequency and low-frequency features contribute to differentiation between PYR and PV cells, allowing near-perfect performance.

### Transforming multichannel spike waveforms to event-based δ-like functions removes all waveform-based information and allows extracting purely spatial features

Having established a cross-validated pipeline for cell type classification from spike data, we turned to focus on spatial features. To limit the information to spatiotemporal features per se, we first removed all single-channel waveform information from the waveforms recorded over the eight channels using an event-based δ-transformation (Materials and Methods; Fig. 3*A*). The procedure was applied to the following three distinct events: the FMC, the NEG, and the SMC. The transformation yielded nearly identical main channel waveforms for all units (Fig. 3*B*). To determine whether the δ-transformation indeed removed all single-channel waveform information, we used the transformed spikes as input for waveform-based feature extraction, followed by model training and testing. The classification models yielded chance-level results. Specifically, the AUC was 0.5 [0.5–0.5] (*p *=* *0.99, Wilcoxon test; Fig. 3*C*). When using a naive decision threshold of 0.5, the same class was predicted for every sample (Fig. 3*C*, inset). Thus, the δ-transformed waveforms are devoid of waveform-based information.

From the transformed waveforms recorded on the eight channels, *n* = 18 spatial features were derived for every unit (Table 3). The features were partitioned into the following three families: time based (Fig. 3*D*), graph based (Fig. 3*E*), and value based (Fig. 3*F*; Materials and Methods). Ten of eighteen (56%) of the spatial features exhibited differences between the PYR and PV populations (*p *<* *0.05, *U* test). To estimate feature redundancy, we computed rank CCs and MI between every pair of spatial features. The matrix of CCs between spatial features (Extended Data Fig. 3-1) showed absolute correlations (median [IQR]: 0.2 [0.1–0.33]) that were smaller than for the waveform-based features (0.43 [0.2–0.77]; *p *=* *3.5 × 10^−4^, *U* test) and for the spike-timing features (0.52 [0.38–0.7]; *p *=* *1.1 × 10^−7^). Furthermore, MI values between spatial feature distributions were smaller than MI between waveform-based features (*p *<* *7.1 × 10^−9^, *U* test) and smaller than MI between spike-timing features (*p *<* *4.3 × 10^−9^). Moreover, absolute intermodality CCs [0.13 (0.07–0.20)] were smaller than absolute intramodality CCs (0.244 [0.13–0.48]; *p *=* *3.1 × 10^−19^, *U* test). The weak correlations between spatial and waveform-based features, and between spatial and spike-timing features, suggest that a combination of features from different modalities may be beneficial for classification. Finally, the differences between the PYR and PV groups for most spatial features, together with the relatively weak mutual information between pairs of spatial features, suggest that the various spatial features may contain nonoverlapping information useful for classification.

### The variance of spatial features over channels and across chunks is different for PYR and for PV cells

**Figure 4.**
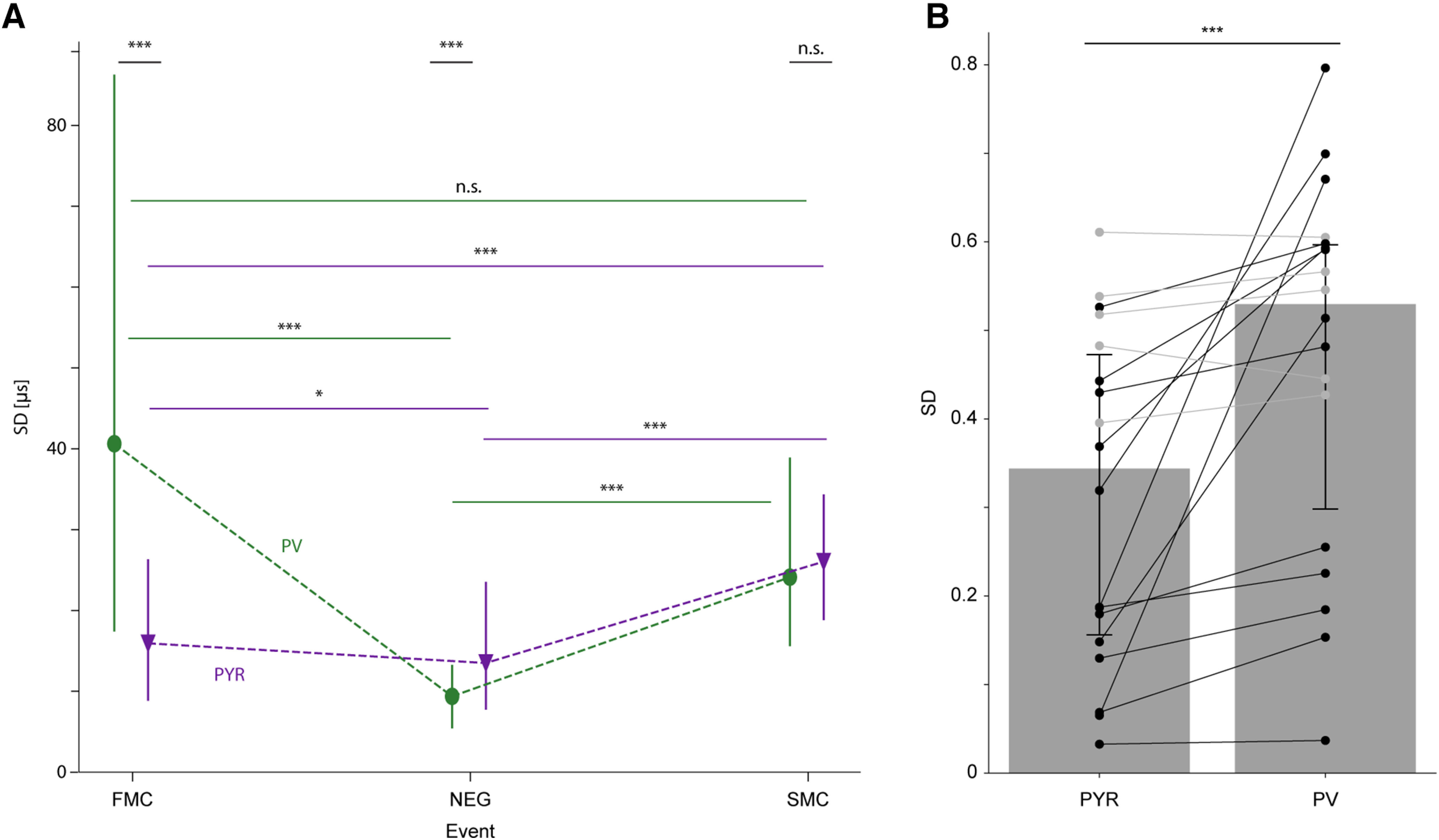
The variance of spatial features over channels and across chunks is different for PYR and for PV cells. ***A***, Variance over channels differs between events and cell types. Compared with PYR, PV cells show lower spatiotemporal spike synchrony (i.e., higher SD) during FMC. The relation reverses during the NEG event. The SD of the SMC event is not consistently different between PYR and PV cells. n.s.*p *>* *0.05, ***p <* *0.001, *U* test. For both PYR and PV cells, synchrony increases from the FMC to the NEG, and then decreases during the SMC. Lined *p < 0.05, ***p < 0.001, Kruskal–Wallis test, corrected for multiple comparisons. ***B***, Variance across chunks differs between cell types. Every dot shows the SD value for a different spatial feature, based on 25 spike chunks. Of 18 features, 13 (72%) differ in the SD values between the cell type groups, with the SD being higher for PV cells (black lines, *p* < 0.05; gray lines, *p* > 0.05, *U* test). Comparing the median SDs of the 18 spatial features between the cell type groups, PV cells exhibit higher SDs compared with PYR. Bars (error bars) represent the median (IQR) of the median feature values for each cell type. ***p < 0.001, two-tailed Wilcoxon test. See also Extended Data Figure 4-1.

A direct comparison of the spatiotemporal dispersion of the event times between PYR and PV cells revealed event-dependent synchronization differences for both cell types (*p *<* *4.5 × 10^−20^, Kruskal–Wallis test; Fig. 4*A*). For PYR, an increase in spatiotemporal synchronization from FMC to NEG was observed (exhibited by a decrease in the SD; FMC: 15.9 μs [8.8–26.3 μs]; NEG: 13.5 μs [7.7–23.5 μs]; *p *<* *0.02, Kruskal–Wallis test, corrected for multiple comparisons). An increase from FMC to NEG was also seen for PV cells (FMC: 40.6 μs [17.4–86.3 μs]; NEG: 9.4 μs [5.4–13.3 μs]; *p *<* *1 × 10^−19^). For both cell types, the increase in synchronization was followed by a decrease from NEG to SMC (SMC: PYR, 26 μs [18.8–34.3 μs], *p *<* *2.2 × 10^−16^; PV, 24.1 μs [15.6–38.9 μs], *p *<* *3.6 × 10^−11^). Thus, for both PYR and PV cells, spatiotemporal synchronization changes during the course of an action potential.

10.1523/ENEURO.0265-22.2022.f4-1 Figure 4-1 Extended Data for Figure 4. Time-lag-SS and Shortest-path features across events and cell types. ***A***, Time-lag-SS features differ between events and cell types. Compared with PYR cells, PV cells show larger feature values during FMC. The relation reverses during the NEG event. The values during the SMC event are not consistently different between PYR and PV cells. For PV cells, feature values decrease from the FMC to the NEG, and then increase during the SMC. For PYR cells, feature values increase between FMC and SMC, and between NEG and SMC. Here and in ***B***, all conventions are the same as in Figure 4*A*. ***B***, The graph-based shortest-path feature differs between events and cell types. Compared with PYR cells, PV cells exhibit smaller feature values during FMC. The relation reverses during the NEG event. The values during the SMC event are not consistently different between PYR and PV cells. For PV cells, feature values increase from the FMC to the NEG, and then decrease during the SMC. For PYR cells, feature values decrease between FMC and SMC, and between NEG and SMC. Download Figure 4-1, TIF file.

Next, we assessed whether PYR and PV cells exhibit differences in spatiotemporal synchronization during specific events. Higher spatiotemporal synchronization was observed for PYR spikes compared with PV cells during FMC, expressed by lower SD (A_w_ = 0.72; *p *=* *2.3 × 10^−12^, *U* test; Table 3). Thus, the FMC event occurred on multiple channels nearly at the same time for PYR spikes, whereas for PV cell spikes the FMC was more dispersed in time. Synchronization flipped during the NEG event, with higher synchronization for the PV cell spikes (Aw = 0.63; *p *=* *3.44 × 10^−5^, *U* test). We did not observe consistent differences during the SMC event (Aw = 0.50; *p *=* *0.47, *U* test). Similar changes between events were observed for the Time-lag-SS and the Shortest-path features (Extended Data Fig. 4-1). The synchronization differences between the spikes of PYR and PV cells may reflect the distinct morphologic and functional properties of the different cell types.

Intraunit variability, the variability across the chunks of the same unit, may degrade classification performance. Alternatively, intraunit variability may differ between classes and possibly benefit classification. Of the statistics extracted from the chunks, the SD is a second moment statistic, and may hold unique information compared with the other chunk statistics used. Specifically, we examined the intraunit SD values for all spatial features calculated based on 25 spike chunks (the smallest chunk size used). To allow comparing SDs of multiple features, features were scaled based on all units before calculating the SD for every unit. Most features (13 of 18) showed consistent differences of the SD between the PYR and PV cells groups (0.56 ≤ A_w_ ≤ 0.90; *p* < 0.05, *U* test; Table 4). All features that did not differ consistently between the two cell types were of the graph-based family (FMC-Average-weight, SMC-Average-weight, SMC-Longest-path, FMC-Shortest-path, and SMC-Shortest-path; 0.50 ≤ A_w_ ≤ 0.54; *p *>* *0.05, *U* test; Fig. 4*B*, gray lines). All the features that consistently differed between the two cell types exhibited larger SD values for PV cells, compared with PYR (Fig. 4*B*, black lines). The median SDs for all features were lower for PYR (0.34 [0.16–0.47]) compared with PV cells (0.53 [0.3–0.6]; *p *= 1.9 × 10^−4^, Wilcoxon test). The higher intraunit variability for PV cells indicates a common phenomenon of the spatial features that is identified specifically by chunking.

**Table 4:** SD across Chunks for spatial features

Feature	Event	PYR SD (scaled) median [QR]^*a*^	PV SD (scaled) median [IQR]^*a*^	A_w_^*b*^	*p*-value^*c*^
Time-lag-SS	FMC	0.065 [0.019–0.22]	0.67 [0.42–0.93]	0.90	8.9 × 10^−37^
	NEG	0.033 [0.011–0.076]	0.037 [0.011–0.21]	0.56	0.022
	SMC	0.15 [0.097–0.23]	0.51 [0.25–0.83]	0.83	1.1 × 10^−25^
Time-lag-SD	FMC	0.19 [0.093–0.41]	0.80 [0.61–0.94]	0.90	1.3 × 10^−35^
	NEG	0.19 [0.10–0.32]	0.23 [0.12–0.48]	0.56	0.02
	SMC	0.32 [0.26–0.42]	0.70 [0.50–0.93]	0.85	1.6 × 10^-27^
Average weight	FMC	0.61 [0.36–0.84]	0.61 [0.41–0.76]	0.50	0.46
	NEG	0.43 [0.28–0.62]	0.48 [0.36–0.68]	0.57	0.016
	SMC	0.54 [0.43–0.65]	0.57 [0.40–0.70]	0.53	0.2
Longest path	FMC	0.53 [0.31–0.81]	0.60 [0.43–0.86]	0.57	0.016
	NEG	0.44 [0.29–0.68]	0.59 [0.37–0.76]	0.60	0.0015
	SMC	0.52 [0.34–0.72]	0.55 [0.36–0.75]	0.52	0.22
Shortest path	FMC	0.48 [0.22–0.92]	0.45 [0.33–0.67]	0.50	0.47
	NEG	0.37 [0.15–0.71]	0.59 [0.27–0.86]	0.65	7.2 × 10^−5^
	SMC	0.40 [0.26–0.60]	0.43 [0.28–0.68]	0.54	0.097
SPD-Count		0.069 [0–0.35]	0.15 [0.037–0.40]	0.60	0.0013
SPD-SD		0.18 [0.12–0.24]	0.26 [0.19–0.30]	0.68	6.3 × 10^−9^
SPD-Area		0.13 [0.10–0.18]	0.18 [0.15–0.24]	0.73	1.1 × 10^−12^

a Based on 25 spike chunks.

b A_w_ ranges from 0.5 (no difference) to 1 (nonoverlapping distributions).

c Mann-Whitney *U* test.

### Features based exclusively on spatial information allow accurate classification of PYR and PV cells

**Figure 5.**
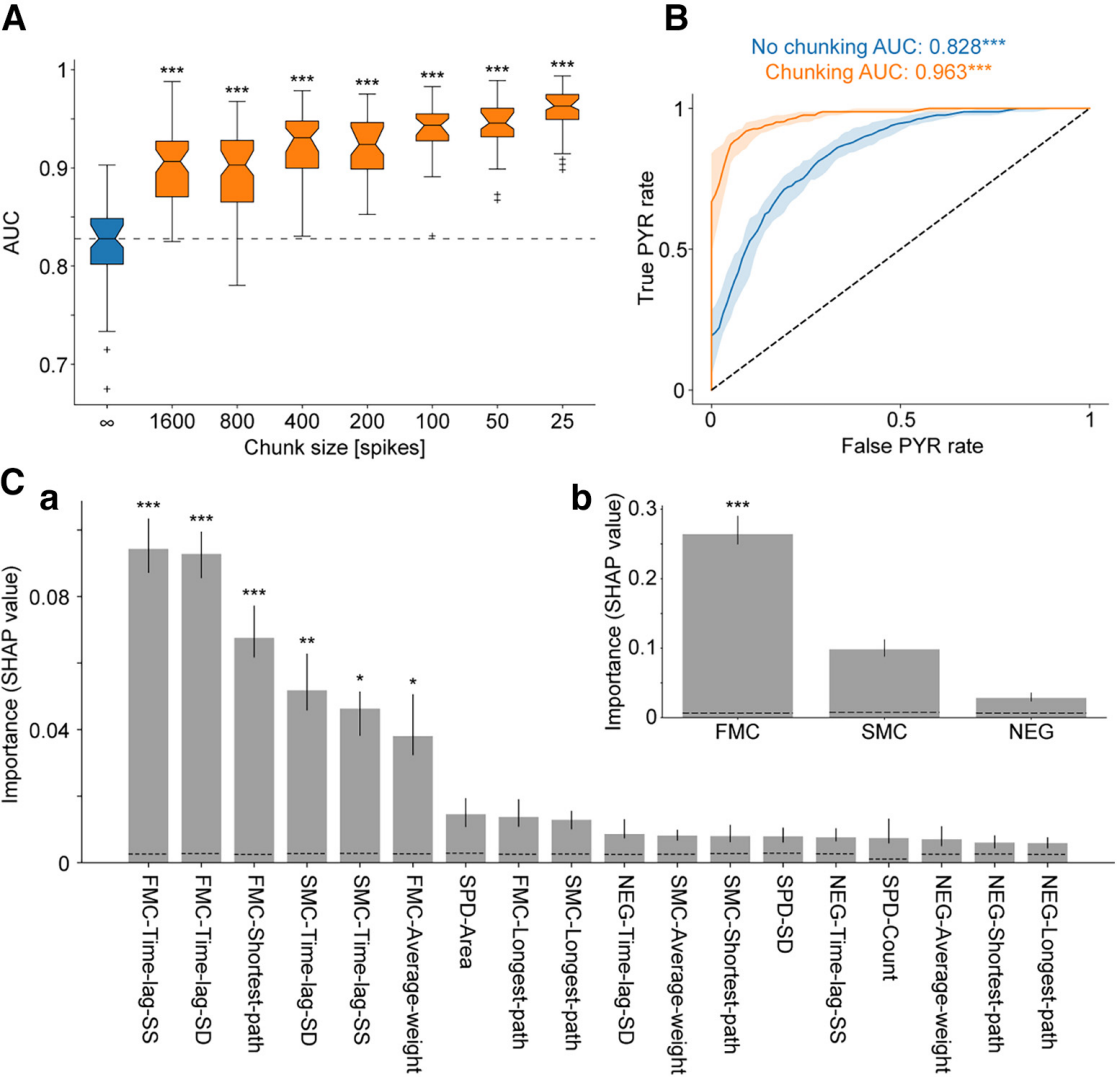
Features based exclusively on spatial information allow accurate classification of PYR and PV cells. ***A***, Classification based on spatial features is boosted by chunking. AUCs were derived from ROC curves based on *n* = 50 cross-validated random forest models. The AUC increases monotonically when chunk size is reduced. Every boxplot shows the median (IQR), whiskers extend for 1.5 times the IQR in every direction, a plus indicates an outlier, and notches represent 95% confidence intervals based on bootstrapping with 1000 repetitions. The best performance (highest AUC) and largest improvement compared with no-chunking (∞) is observed for 25 spike chunks. ***p <* *0.001, Wilcoxon test. ***B***, Spatial features allow accurate classification. ROC curves for the test data without chunking (blue) and with 25 spike chunks (orange). The AUCs are higher than chance level. All conventions are the same as in Figure 2*B*. ***C***, Feature importance analysis for spatial models with 25 spike chunks. SHAP values were used to assess the individual contribution of each feature to the prediction. The dotted lines represent chance-level importance values, based on models trained with shuffled PYR-PV labels. The features derived from the FMC event are associated with the highest SHAP values, indicating that synchrony at the initial depolarization phase makes the highest contribution to classification outcome. **p <* *0.01, ***p <* *0.001, one-tailed permutation test. See also Extended Data Figure 5-1.

To determine whether the differences between the spatial distribution of the extracellular signals from PYR and PV cells are only correlative or indicative, we conducted a training and evaluation process. The process was carried in the same manner as for the waveform-based and spike-timing features. Using the mean waveforms, the median [IQR] AUC was 0.83 [0.8–0.85] (*p *=* *3.8 × 10^−10^, Wilcoxon test; Fig. 5). The performance of the spatial models improved when chunking was applied: chunking consistently increased the performance for all tested chunk sizes (25–1600 spikes: *p *<* *1.45 × 10^−9^, Wilcoxon test; Fig. 5*A*). Upon chunking to 1600 spike chunks, the AUC increased by 9.6% compared with the no-chunking AUC. The AUC increased monotonically for progressively smaller chunk sizes, achieving a value of 0.963 [0.949–0.975] for 25 spike chunks (16.3% [13.9–19.6%] increase; Fig. 5*B*). We did not test smaller chunk sizes. Therefore, results reported from this point onward are based on the best model, using 25 spike chunks. While the performance of the spatial models was lower than the performance of waveform-based or spike-timing models (*p *<* *6.6 × 10^−6^ for both, two-tailed Wilcoxon test), the results indicate that PYR and PV cells can be accurately differentiated based on purely spatial features. On their own, spatial properties provide a completely new approach to cell type classification.

10.1523/ENEURO.0265-22.2022.f5-1 Figure 5-1 Extended Data for Figure 5. Distribution of the six most important spatial features. ***A***, Cumulative distributions of the FMC-Time-lag-SS feature calculated without chunking. Here and in all subsequent cumulative distribution functions, horizontal lines represent 50%, vertical dashed lines indicate medians. n.s.*p *>* *0.05; ***p <* *0.001, *U* test. ***B***, Cumulative distributions of the FMC-Time-lag-SD feature. ***C***, Cumulative distributions of the FMC-Shortest-path feature. ***D***, Cumulative distributions of the SMC-Time-lag-SD feature. ***E***, Cumulative distributions of the SMC-Time-lag-SS feature. ***F***, Cumulative distributions of the FMC-Average-weight feature. Download Figure 5-1, TIF file.

To assess the contribution of every spatial feature and family of features, we analyzed SHAP values. We found that feature importance was not uniform (Fig. 5*C*). Specifically, the highest importance was attributed to features derived from the FMC events (median [IQR] over all 50 instantiations: FMC-Time-lag-SS: 0.094 [0.087–0.1]; FMC-Time-lag-SD: 0.093 [0.086–0.099]; *p *<* *0.001 for both, permutation test; Fig. 5*C*). The feature families differed in the contribution to the prediction (*p *<* *1.7 × 10^−29^, Kruskal–Wallis test). Features of the value-based family exhibited the lowest summed importance values (0.028 [0.021–0.036]), while the two other families reached higher values (time-based: 0.25 [0.23–0.27], graph-based: 0.11 [0.1–0.13]; *p *=* *2.6 × 10^−8^ for both, Kruskal–Wallis test corrected for multiple comparisons). The importance of the time-based features was the largest (time based compared with graph based, *p *=* *2.6 × 10^−8^). The distribution of the six most important features is shown in Extended Data Figure 5-1. The feature importance analysis suggests that features agnostic to the physical distance between channels and features that do consider spatial locations make nonoverlapping contributions to classification. Moreover, the usage of multiple features derived from the same event, the FMC, is beneficial.

### Spatial models generalize poorer than waveform models but better than spike-timing models

**Figure 6.**
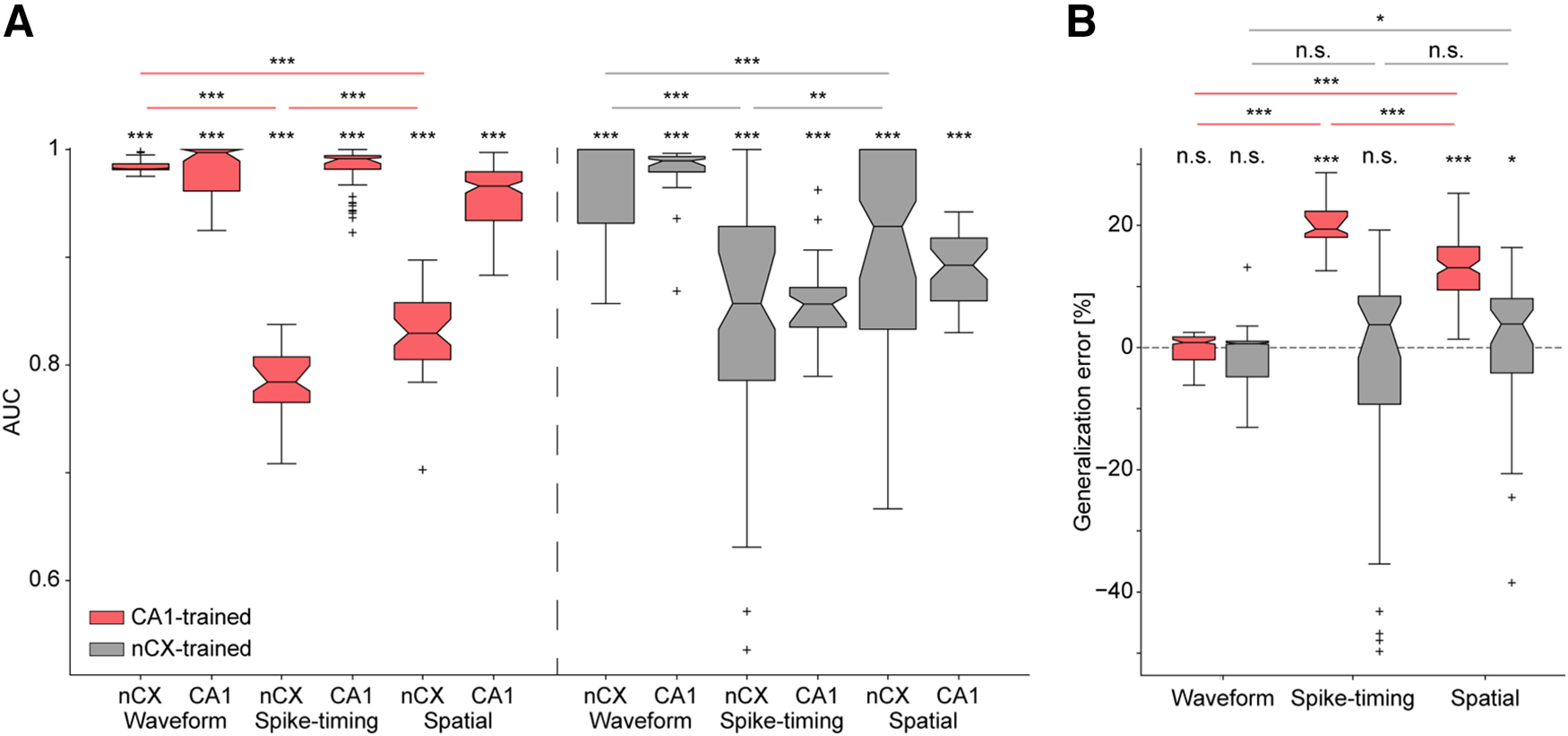
Spatial models generalize poorer than waveform models, but better than spike-timing models. ***A***, Cross-validated random forest models (*n* = 50) were trained for every modality on the CA1 (left, red) or neocortex (nCX; right, gray) data, and tested separately on different data from CA1 and from nCX. Conventions for boxplots here and in ***B*** are the same as in Figure 5*A*. All models exhibit above-chance performance. ***p <* *0.001, Wilcoxon test. The performance of models on the non-trained-on region is highest for waveform-based models and lowest for spike-timing models. **p <* *0.01; ***p <* *0.001, Kruskal–Wallis test corrected for multiple comparisons. ***B***, The decrease in performance on generalization. Generalization error is defined here as the difference between the AUC on the test set of the training region and the AUC on the test set of the non-trained-on region, divided by AUC on the test set of the training region. Spatial models trained on either region and spike-timing models trained on CA1 data, show generalization errors larger than zero. n.s.*p *>* *0.05; **p <* *0.01; ***p <* *0.001, Wilcoxon test. The dashed horizontal line represents the zero-mark (i.e., same performance on CA1 and nCX). When trained on CA1 data (red), spatial models generalize poorer than waveform models but better than spike-timing models. n.s.*p* > 0.05; *p <* *0.05; ***p <* *0.001, Kruskal–Wallis test corrected for multiple comparisons. See also Extended Data Figures 6-1, 6-2.

In the tagged dataset, units were recorded from both CA1 and neocortex, allowing the testing of interregion generalization. To quantify similarities between regions, we determined the performance of models trained using data from a single region on one test set from the training region, and on another test set from the non-trained-on region. Training on either CA1 data or neocortical data, all models performed above chance level when tested on both the CA1 test set and the neocortical test set (*p *<* *7.5 × 10^−10^ for all comparisons, Wilcoxon test; Fig. 6*A*, Extended Data Fig. 6-1). Specifically, spatial models trained on CA1 data reached a median [IQR] AUC of 0.966 [0.934–0.979] when tested on CA1 data and 0.83 [0.805–0.858] when tested on neocortical data. Complimentarily, when trained on neocortical data, spatial models reached an AUC of 0.923 [0.833–1] on the neocortical test set, and an AUC of 0.893 [0.86–0.918] on the CA1 test set. Comparing AUCs of the non-trained-on region, performance was lower for the spike-timing models compared with the waveform-based models (*p *<* *2.2 × 10^−16^ for both comparisons, Kruskal–Wallis test corrected for multiple comparisons; Fig. 6*A*). Likewise, performance was lower for the spike-timing models, compared with the spatial models (*p *< 0.005 for both comparisons; Fig. 6*A*). Thus, while all modalities generalize from CA1 to neocortex and from neocortex to CA1, waveform-based models allow the best performance whereas spike-timing models perform the worst.

10.1523/ENEURO.0265-22.2022.f6-1 Figure 6-1 Extended Data for Figure 6. Models of all modalities generalize between brain regions. ***A***, Models based on waveform-based features (50 spike chunks) were trained on CA1 data (top) or on neocortical data (bottom). The AUCs were calculated based on *n* = 50 models. Performance of waveform-based models is above chance level when tested on either CA1 (left) or neocortical (right) samples. Here and in ***B*** and ***C***, ***p <* *0.001, Wilcoxon test. ***B***, Models based on spike-timing features (1600 spike chunks) were trained on CA1 data (top) or on neocortical data (bottom). Performance of spike-timing models is above chance level when tested on either CA1 or neocortical samples. ***C***, Models based on spatial features (25 spike chunks) were trained on CA1 data (top) or on neocortical data (bottom). Performance of spatial models is above chance level when tested on either CA1 or neocortical samples. Download Figure 6-1, TIF file.

Above-chance performance on the test set of the non-trained-on region does not guarantee perfect generalization. To quantify generalization, we defined a “generalization error” as the relative decrease when comparing performance on the test set of the training region and performance on the test set of the non-trained-on region. The generalization error was consistently above zero for spatial models trained on data from either region, and for CA1-trained spike-timing models (*p *<* *0.05 for all three comparisons, Wilcoxon test; Fig. 6*B*). For both training sets, waveform-based models showed lower errors than spatial models (*p *<* *0.05 for both comparisons, Kruskal–Wallis test corrected for multiple comparisons; Fig. 6*C*). In addition, for the CA1-trained models, the generalization error of the spatial models was lower than that of the spike-timing models (*p *<* *6.7 × 10^−6^). Thus, spatial models generalize better than spike-timing models, but worse than waveform-based models, in particular when trained on CA1 data.

Finally, to determine which spatial features are most important for classification in every region, we computed SHAP values for spatial models trained on data from a single region. Despite some differences in specific values, the six features that made the largest contributions were the same for models trained on CA1 data (Extended Data Fig. 6-2*A*) and for models trained on neocortical data (Fig. 5*C*, Extended Data Fig. 6-2*B*). Hence, the determinants for the predictions of the spatial models are similar in neocortical and CA1 data.

10.1523/ENEURO.0265-22.2022.f6-2 Figure 6-2 Extended Data for Figure 6. Spatial feature importance indicates consistent characteristics across regions. ***A***, SHAP values for the spatial models with 25 spike chunks trained only on CA1 data. The six most important features are the same as the six most important features in the analyses of models trained on the data from both regions (Fig. 5C). ***B***, SHAP values for the spatial models with 25 spike chunks trained on nCX data. The SMC features are the strongest determinants of the predictions for neocortical-trained models. The six most important features are the same as for the CA1-trained data (***A***) and as for the models trained on the data from both regions (Fig. 5*C*). Download Figure 6-2, TIF file.

## Discussion

Using optically tagged high-density recordings from hippocampal region CA1 and neocortex of freely moving mice, we found that spiking of PYR and PV cells was associated with different spatiotemporal distributions of extracellular voltage. Compared with PV cell spikes, PYR spikes exhibited higher spatial synchrony at the beginning of the spike and lower synchrony at the trough. Together, the spatial features derived from the extracellular voltage distributions allowed accurate classification of PYR and PV cells.

### Differences in the spatial distribution of extracellular voltages during spikes

Although the contribution of spatial information to classification tasks has been explored before (Buccino et al., 2018; Jia et al., 2019), previous work did not separate spatial information from other waveform-based properties. Using an event-based δ-transformation, we derived spatial features devoid of single-channel waveform information. The accurate classification based on spatial features is tantamount to spatiotemporal differences in the extracellular voltage distribution and is consistent with morphologic differences in the dendrosomatic and axonal organization of PYR and PV cells. In CA1, PV basket cell axons form a diverse horizontal network while most dendrites extend vertically (Freund and Buzsáki, 1996; Klausberger et al., 2003). The dendritic trees of PYR cells also extend vertically, but are more polarized (Bannister and Larkman, 1995; Spruston, 2008). The extracellular expression of intracellular signals has been studied theoretically (Rall, 1962) and modeled for reconstructed morphologies (Holt and Koch, 1999; Gold et al., 2006; Schomburg et al., 2012; Lindén et al., 2014; Bestel et al., 2021). Even with limited spatial sampling, the present results provide direct experimental evidence for a unique mapping between cell type and the spatial distribution of extracellular potentials. The results should be construed as lower bounds, since higher-density or three-dimensional sampling may allow further improvement.

Among spatial features, the dispersion of the FMC event between recording sites made the highest contribution to the prediction. The FMC is a putative extracellular analog of the initial depolarization phase at the recording site. Thus, the lower interelectrode variance of FMC among PYR cells compared with PV cells indicates higher spatial synchrony at the beginning of the spike. This observation is consistent with known morphologic and electrotonic differences between the proximal dendrites of PYR and PV cells. Since the probes were always inserted perpendicularly to CA1 stratum pyramidale, the vertical dendritic tree of both PYR and PV cells was parallel to the extracellular electrode arrays. In CA1, PYR have thick apical dendrites extending for up to 250 μm (diameter, 1.6–2.5 μm), whereas PV cell dendrites in stratum pyramidale are thinner (diameter, 1.3–1.7 μm; Gulyás et al., 1999; Andersen et al., 2007). The higher FMC synchrony of PYR spikes is consistent with lower axial resistance of the thicker proximal PYR dendrites, yielding synchronized somatic and dendritic potentials.

The second event that showed differences in temporal dispersion was the NEG, which corresponds to a point between the peak of the derivative and the peak of membrane potential during the spike (Henze et al., 2000; Pettersen and Einevoll, 2008). During NEG, spatial synchrony reverses, being higher for PV cells compared with PYR cells. The narrower waveforms of PV cells are associated with a higher concentration of voltage-gated K^+^ (Kv) channels, compared with PYR cells (Bean, 2007). In neuronal models with passive dendrites and when membrane time constants are relatively slow, membrane resistance has little effect on spike shape (Pettersen and Einevoll, 2008). However, since active conductance affects spike shape (Martina and Jonas, 1997), the differences in synchrony during the NEG event may result from distinct spatial gradients of Kv channels in CA1 PYR and PV basket cells. In PYR, there is a rapid decrease in the density of Kv channels on dendrites farther from the soma (Johnston et al., 2000), whereas the decrease for basket cells is more moderate (Hu et al., 2010). Kv channels open at depolarization, initiating repolarization and late hyperpolarization, and, in turn, govern the frequency and width of the spikes (Pongs, 1999). The more uniform spatial density of Kv channels in PV cells compared with PYR cells may synchronize the extracellular signals generated by the different cellular compartments.

The spatial features exhibit lower intraunit variability of PYR cells, compared with PV cells. The difference between the PYR and PV variability contributed to classification. The excitatory input of PYR in CA1 originates mainly from upstream regions (e.g., CA3 and entorhinal cortex; Andersen et al., 2007), whereas CA1 PV cells are mainly innervated by local PYR cells (Freund and Buzsáki, 1996). Thus, a possible source for the difference in intraunit variability is the distinct sources of excitation of PYR and PV cells.

### Differences in spike waveform and spike timing

Compared with basket cells, neocortical and CA1 PYR have wider spikes (Simons, 1978; Connors et al., 1982; Kawaguchi and Hama, 1988; Contreras, 2004), lower firing rates (Kawaguchi et al., 1987), and an increased burst propensity (Kandel and Spencer, 1961; Ranck, 1973; Harris et al., 2001). Indeed, we found that waveform width (e.g., TTP-duration), burstiness (e.g., Uniform-distance), and firing rate all differ between PYR and PV cells. Furthermore, both TTP-duration in the waveform models, and the Uniform-distance in the spike-timing models, contributed to the prediction. The importance of waveform width properties is in line with studies that used width-related features to differentiate between PYR and interneurons (Frank et al., 2001; Cardin et al., 2009; Stark et al., 2013). Similarly, the importance of burstiness and firing rate is consistent with prior work (Connors and Gutnick, 1990; Taira and Georgopoulos, 1993). The long-term ACH, which was not used before for classification, held informative value, consistent with distinct low-frequency rhythmic activity of PYR and PV cells (e.g., theta; Csicsvari et al., 1999; Buzsáki, 2002; Czurkó et al., 2011).

### Chunking

When discussing the extracellular waveform of a neuron, many studies refer to the mean waveform (Trainito et al., 2019; Sun et al., 2021). To improve the performance of the classification models, we exploited the variability of spike waveforms and timing recorded from a single cell using chunking, increasing the number of samples at the possible cost of increased noise. The chunking method as implemented here was agnostic to two pieces of information. First, spikes were randomly assigned to chunks, ignoring possible time-related changes that may be constructive. Second, the relation of chunks to the same unit was only partially considered, and additional “chunk statistics” may be extracted. The statistics extracted from the distribution of feature values over chunks provide limited consideration of the other chunks. Consequently, the present implementation does not allow classification of all the chunks of a specific unit as a whole. Hence, our results form a lower bound for the improvement to be gained from chunking. More complex models may capitalize on time-dependent differences and dependencies between the samples. Modifying chunk size inherently results in a trade-off between the number of samples and the noise. Spatially and waveform-based models benefited the most from smaller chunks, while spike-timing models benefited from larger chunk sizes. The higher sensitivity of spike-timing models to noise in small chunks is consistent with the discrete nature of the spike trains, because ACHs are sparse when the number of spikes is small.

### Interregion differences

Waveform models yielded near-perfect classification of data from the non-trained-on region for both training regions, in line with similar waveform widths of PYR and PV cells in neocortex and CA1 (McCormick et al., 1985; Kawaguchi and Hama, 1988). Yet, the generalization of waveform models was not perfect and is not expected to be universal: in the primate, pyramidal tract PYR cells exhibit narrow spikes (Vigneswaran et al., 2011; Lemon et al., 2021). CA1-trained spike-timing and spatial models exhibited decreased performance when tested on neocortical data compared with CA1 data, with spatial models generalizing better than spike-timing models. The poor generalization observed for the spike-timing models is consistent with the fact that neocortical PYR cells are less likely to exhibit bursts, compared with CA1 PYR cells (McCormick et al., 1985; Lacaille et al., 1987). The intermediate generalization of the spatial models may correspond to interregion differences in cellular morphology, ion channel distributions, or other cellular network properties. The synaptic and intracellular events that occur just before and during the spike may affect the spatial distribution of the signal (Zador et al., 1995; Hagen et al., 2016, 2017). Hence, even morphologically identical cells with the exact same compartmental distribution of ion channels are expected to show different spatial distributions of extracellular potentials when embedded in distinct networks.

### Limitations and applications

There are a few notable limitations to this work. First, cell type classification based on spatial features requires sampling of the extracellular space over multiple points. Here, we used a fixed electrode configuration with 20 μm vertical spacing, and application to data recorded using other configurations may require modifications. Second, expanding the duration of the sampled spikes beyond 1.6 ms (32 samples at 20 kHz) may increase the performance of spatially based models. Third, while several models yielded near-perfect performance, focusing strictly on mice does not warrant generalization to homological brain regions in other animals.

Our results suggest several possible applications. First, the concept of chunking combined with a majority vote can be used in real time, allowing classification outcomes to be updated online. Using the chunking and voting approach, there is no need to rerun the entire model whenever a new piece of data is collected. Instead, every time a predetermined number of spikes is gathered, another vote can be added to the prediction. Second, classification based strictly on spike timing can be used when waveform information is unavailable or for real-time applications.

### Future directions

To identify the cellular network origin of the spatiotemporal differences in synchrony between PYR and PV cells, targeted experiments may be conducted. We hypothesize that the higher intraunit variability observed for PV cells compared with PYR cells may reflect distinct connectivity patterns. The excitatory input of PYR cells in CA1 originates mainly from upstream regions (e.g., CA3 and entorhinal cortex; Andersen et al., 2007), whereas CA1 PV cells are more likely to be innervated by local PYR cells (Freund and Buzsáki, 1996). The hypothesis may be tested using somatic opsins (Shemesh et al., 2017; Chen et al., 2018; Forli et al., 2021). The spatiotemporal distribution of extracellular potentials during spikes generated via somatic activation can be compared with natural spiking, generated by integrating EPSPs impinging mainly on the dendrites. More consistent spatiotemporal synchrony achieved for optically induced spikes will provide direct evidence that input variability may lead to more variability in the spatiotemporal synchrony.

In the future, other spatial features may be used to increase classification performance. We showed that the cross-validated classification of PYR and PV cells is already near perfect when based on waveforms alone, but other cell types may not be accurately distinguished using features derived from a single channel. For instance, compared with PV cells, somatostatin interneurons have lower firing rates and broader spikes (Ma et al., 2010; Royer et al., 2012; Veit et al., 2017). Distinguishing between multiple cell types using extracellular data may benefit from using spatial information.

## Synthesis

Reviewing Editor: Arvind Kumar, KTH Royal Institute of Technology Decisions are customarily a result of the Reviewing Editor and the peer reviewers coming together and discussing their recommendations until a consensus is reached. When revisions are invited, a fact-based synthesis statement explaining their decision and outlining what is needed to prepare a revision will be listed below. The following reviewer(s) agreed to reveal their identity: Gaute Einevoll. Note: If this manuscript was transferred from JNeurosci and a decision was made to accept the manuscript without peer review, a brief statement to this effect will instead be what is listed below.

Synthesis

Identification of cell types based on extracellular shape of spikes is an important and interesting challenge in neuroscience. In this study authors report that pyramidal cells and PV+ interneurons have distinct spatio-temporal signatures in the extracellular spikes. Overall both the reviewers have appreciated the approach taken in this paper to address this important question. The result that extracelular signatures of pyramidal and PV cells could be seen in the spatio-temporal features rather than the spike shape or spike-timing features is very interesting and potentially useful. But reviewers have also raised several concerns:

Explanation of the findings: In its current form the paper is very descriptive and lacks mechanistic understanding. Ideally to address this may have to resort to computational modelling but that is out of scope of this work. However, based on previous modelling work already cited (and also Pettersen and Einevoll, Biophysical Journal, 2008) authors should be able to provide a testable hypothesis.

Conceptual errors:

extracellular electrodes measure the distribution of electrical potential not distribution of electric fields - this should be corrected both in the title and in the text

the link between the linear correlation and the term “redundancy”: Please note that linear correlations provide only a first order approximation of redundancy. Weak linear correlation does not preclude non-linear correlations. Authors therefore should complement their finding by estimating non-linear correlation (see below for more detailed comment) between distribution of features.

Besides these, reviews have raised a number of concerns that could be considered as minor but have to be addressed in the revised version of the manuscript. Detailed comments from the two reviews are appended below.

# Reviewer 1

The paper of Sukman and Stark addresses an interesting and important subject in neuroscience, namely the identification of cell types, here pyramidal cells vs. PV cells, based on their spikes, that is, their extracellular signatures of action potentials. An experimental approach is used where the ground-truth identification is based on optogenetic tagging of PV cells. The work is comprehensive and seems competently done, and I recommend publication in eNeuro.

However, I have some minor comments the authors should address:

1. On l. 77 it reads: “However, despite extensive modelling, the relationship between the distribution of electrical fields and neuronal morphology, and in particular cells, is not as well understood.”

(a) For one, it is not the distribution of electrical fields that are measured by multi-electrodes, it is the distribution of electrical potentials. The electrical fields are in general given by the gradient of the electrical potentials. This incorrect word use should be corrected, also in the title.

(b) In the paper, the author focuses on the analysis of spikes, that is, extracellular potentials associated with action potentials. However, there are other measured extracellular potentials, for example, local field potentials (LFPs), and the authors should make it clear that these are not considered here.

2. In the Discussion the authors seem to be unaware of the paper by Pettersen & Einevoll, Biophysical Journal, 2008 which explored the link between neuronal morphologies and spike shapes and amplitudes using a combination of numerical simulations and analytical modeling:

(a) on l. 643 It refers to the old rule of thumb that the spike potential is roughly proportional to the derivative of the membrane potential. In frequency space this would mean that the frequency component of the spike is proportional to the frequency component of the membrane potential multiplied by the frequency (f). In Pettersen & Einevoll, 2008 it was instead demonstrated that in fact this proportionality factor is not the frequency (f), but the square root of the frequency (f), see Eq. 22. This corresponds to the spike shape being somewhere in the middle of being proportional to the membrane potential and the derivative of the membrane potential.

(b) on l. 650-652 it is implied that a change in the effective membrane resistance (i.e., more uniform spatial density of Kv channels) should change the width of the spikes. In Pettersen and Einevoll, 2008 it was shown that the membrane resistance seems to have very little effect on the spike shape (given a fixed intracellular action potentials), see Eq. 22.

# Reviewer 2

Comments to the authors:

Sukman et al. provide indirect evidence that the single units of pyramidal and parvalbumin cells exhibit distinct spatiotemporal extracellular electric potentials. The authors combine machine learning tools, statistical analysis, and experimental evidence to investigate the differences between the extracellular signatures of pyramidal and parvalbumin action potentials, embedding such investigation in the framework of a classification task. The work underlies the importance of including spatiotemporal features when classifying extracellularly recorded single units, rather than exclusively relying on waveform or spike-timing features. The work is well crafted, and results and figures are clearly presented. Therefore I recommend publication in eNeuro. I would like however to address the authors some suggestions to improve the quality and the rigor of their analysis.

General consideration about redundancy and feature importance:

I have appreciated throughout the manuscript the correlation analysis the authors have carried out. I find however improper the link between the linear correlation and the term “redundancy” that the authors have extensively used in the article. A lack of linear correlation is indeed only a first-order approximation of a lack of redundancy. Non-linear relationships might exist between the features’ distributions, and linear correlation analysis might fail to detect them. In the manuscript (see for example line 187 or line 470) the authors seem instead to even make a link between weak correlation and information independence. I would therefore suggest the authors, if truly interested in investigating the relationships between their features, to go beyond linear correlation analysis. One non-parametric and very well-established possibility is offered by information theory measures that were proven to capture even non-linear relationships between distributions. The rigor of the conclusions drawn about the redundancy between features might, in my opinion, greatly improve if employing mutual information measures. Moreover, mutual information can go even beyond the bivariate analysis that the authors have carried out. There are several toolboxes available that might help the authors to compute mutual information quantities (such as the redundancy or the synergy) of their extracted features with little effort, see for example Magri et al. 2008 (https://doi.org/10.1186/1471-2202-10-81), Mayner et al. 2018 (https://doi.org/10.1371/journal.pcbi.1006343) or Timme and Lapish 2018 (https://doi.org/10.1523/ENEURO.0052-18.2018).

Furthermore, adding mutual information might also reinforce the conclusions the authors propose about features’ importance, in the manuscript accessed by adopting the SHAP values. Even though I appreciate the correct use of SHAP values in the article, I also think that a second and independent method, such as mutual information, might overcome some limitations of SHAP such as, for example, the possibility of introducing some bias in the estimation of the Shap values when limiting the depth of the trees.

Alternatively (i.e., if the authors don’t agree with me and feel it is unnecessary to add mutual information measures to their analysis) I would recommend adding some comments about the limits of linear correlation for investigating redundancy between features. Moreover, some limitations about the use of SHAP values to access features’ importance in a classification task should be addressed as well.

Some minor comments:

Line 65: Please consider changing the term “functional connectivity” which might be misleading.

Line 80: As I partially disagree with the authors, I would suggest offering a more specific comment about what the authors think is still missing in the characterization of the relationship between the distribution of extracellular electrical potentials and neuronal morphology.

Line 148: It is not clear to me whether the units identified as inhibitory when showing a significant trough in the monosynaptic time range were then included in the dataset and labeled as PV. If this is the case, do the authors think that some of these units might be non-PV?

Line 161: The authors have decided to interpolate the mean waveform of each unit. Have the authors instead considered interpolating the waveforms before the average? Would this affect their result?

Line 178: To what extent do the authors think the TTP duration is influenced by the duration (32 samples) of the considered waveform?

Line 182: Given the high number of samples for the two distributions tested, it is not surprising to see a significant difference in the U-test. I would therefore add some measure of effect size (like Cohen’s d) to get at least a grasp on whether (and how much) the extracted features might be actually useful for the classification.

Line 242: How many channels were discarded from the analysis after the arbitrary threshold on the magnitude of TTP?

Line 273: Could the authors be more specific when stating that “model weights were adjusted to balance the two classes.”?

Line 273: It is not clear to me in which set the authors have performed the hyperparameter grid search. Is it the entire dataset?

Line 309: The statistical procedure used to test the significance of the SHAP values is not thoroughly described. How many shuffles of the dataset were carried out to obtain the null distribution SHAP values? And which statistical procedure was then carried out to assess the significance of the SHAP values? Did the authors test the SHAP values distribution against the 95th percentile of the null distribution? More details are needed in this regard.

Line 373: It is not clear to me how can the authors be sure that the 21 inhibitory neurons found with the CCH analysis are not (even partially) the same set of the optogenetically tagged inhibitory neurons.

Figure 2a: please add the x-label in Fig. 2 Ca top.

Line 466: Would the authors consider reporting the rank correlation matrix? It could be interesting to show to the readers the degree of the linear relationship between the features.

Line 466: It is not clear to me whether the rank correlation matrices were computed separately for the 3 different families of features. If this is the case, do the authors consider it possible to also compute (and report) the inter-family correlations?

Line 514. Please report in the text the range of synchronization during the NEG phase. Furthermore, as previously mentioned, a size effect analysis would improve the rigor of the statistical analysis. My impression from figure 4 is, indeed, that the difference in the SD during the NEG is, although significant, very limited.

Line 514: Please report in the text the range of synchronization during the SMC event.

Line 516: Was any multiple correction strategy considered for this Wilcoxon test?

Line 547: The authors through the SHAP analysis reveal that the shortest path and the time lag SS of the FMC event are the most important features. I think it would be interesting if the authors present, similarly to what was done in Figure 4, the distributions of such features, in order to grasp the real differences between PYR and PV in these “most important” features.

"Spatial models generalize poorer than waveform models but better than spike-timing models”.

I have personally really appreciated this paragraph. It is, however, in my opinion, still incomplete. It would be really interesting, indeed, to replicate the SHAP analysis in this paragraph. This could help the authors to address some unanswered questions, such as: do the feature importance list presented in Figure 5 still be preserved when using the CA1 training set? And what about the neocortical one? Furthermore, could the SHAP analysis help understand (and quantify) which features are most preserved from hippocampal to neocortical single units? I think that some details in this regard are still missing in the current version of the manuscript.
