# Supplementary figures and images for: Cortical Pyramidal and Parvalbumin Cells Exhibit Distinct Spatiotemporal Extracellular Electric Potentials
Source: eNeuro. 2022 Dec 6;9(6):ENEURO.0265-22.2022. doi: 10.1523/ENEURO.0265-22.2022 (PMC9744183; doi:10.1523/ENEURO.0265-22.2022)

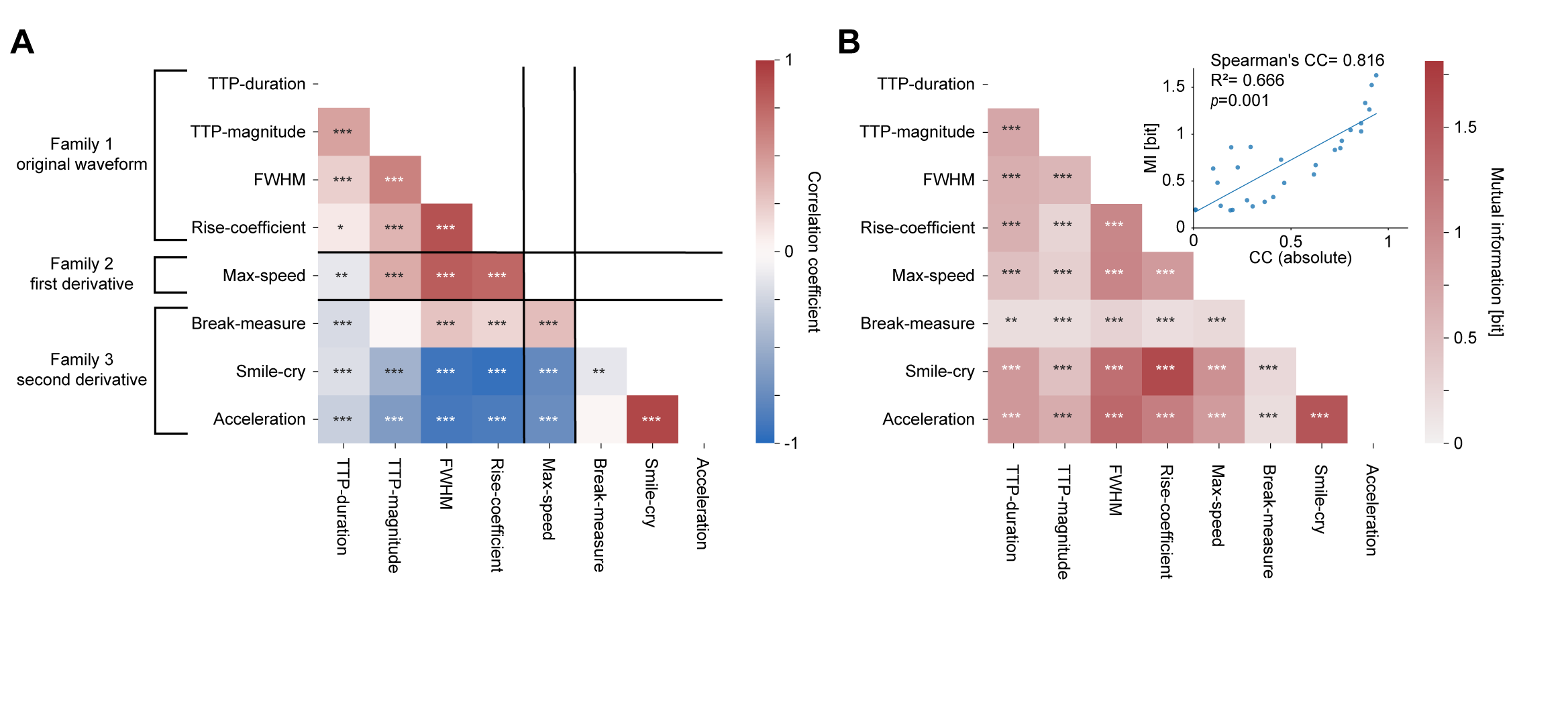

Supplement: Figure 2-1 — Extended data for Figure 2. Waveform-based feature interrelations. A, Rank (Spearman’s) correlations between waveform-based features, grouped by families. Most correlations (26 of 28; 93%) differ from zero. Blank, p > 0.05; *p < 0.05; **p < 0.01; ***p < 0.001; permutation test. B, MI between waveform-based features. All pairs (28 of 28; 100%) exhibit MI values that are higher than chance level. ***p < 0.001, permutation test. Inset, Scatter plot of the MI values between pairs of features and the pairwise absolute rank CCs from A with statistics for rank (Spearman’s) correlation. Download Figure 2-1, TIF file. [file enu-eN-CFN-0265-22-s02.tif]

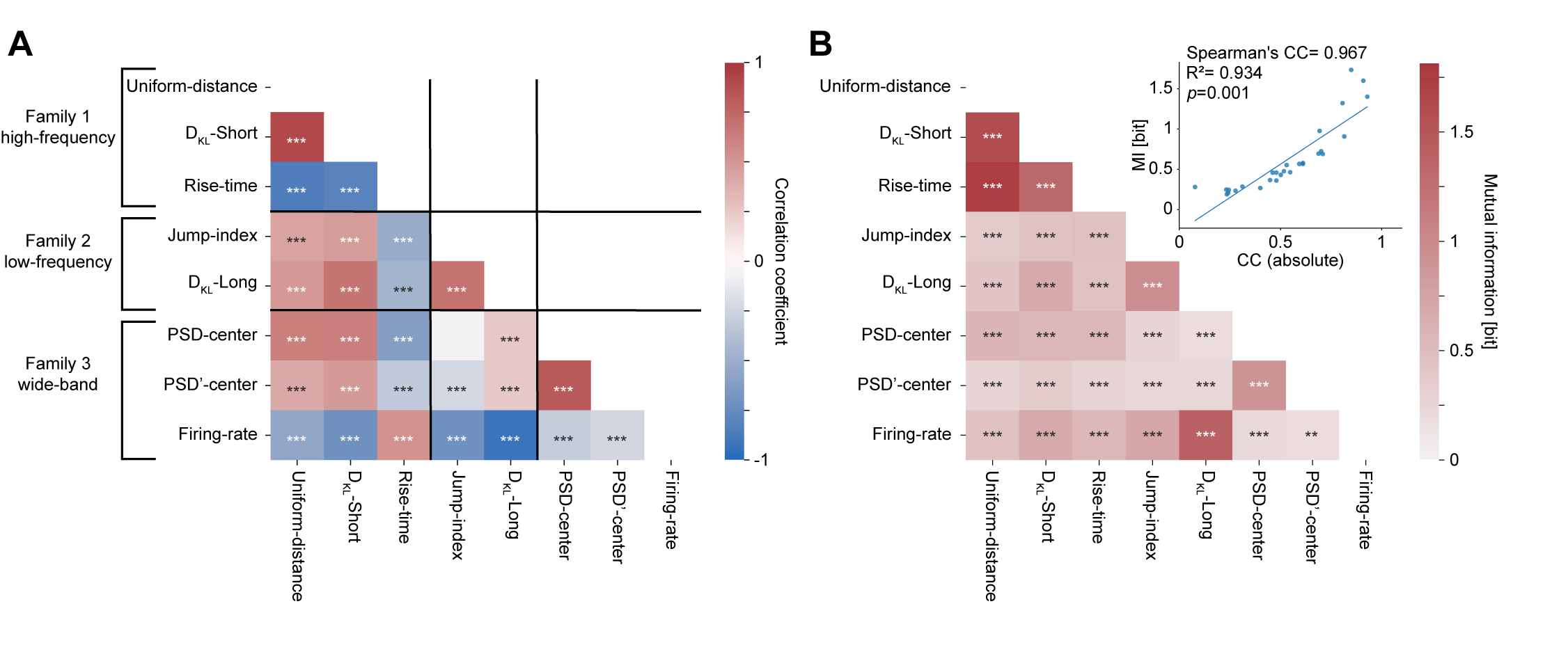

Supplement: Figure 2-2 — Extended Data for Figure 2. Spike-timing feature interrelations. A, Rank correlations between the spike-timing features grouped by families. Most correlations (27 of 28; 96%) differ from zero. All conventions here and in B are the same as in Extended Data Figure 2-1. B, MI between spike-timing features. All pairs (28 of 28; 100%) exhibit MI values that are higher than chance level. Inset, Scatter plot of the MI between pairs of features and the CCs from A. Download Figure 2-2, TIF file. [file enu-eN-CFN-0265-22-s03.tif]

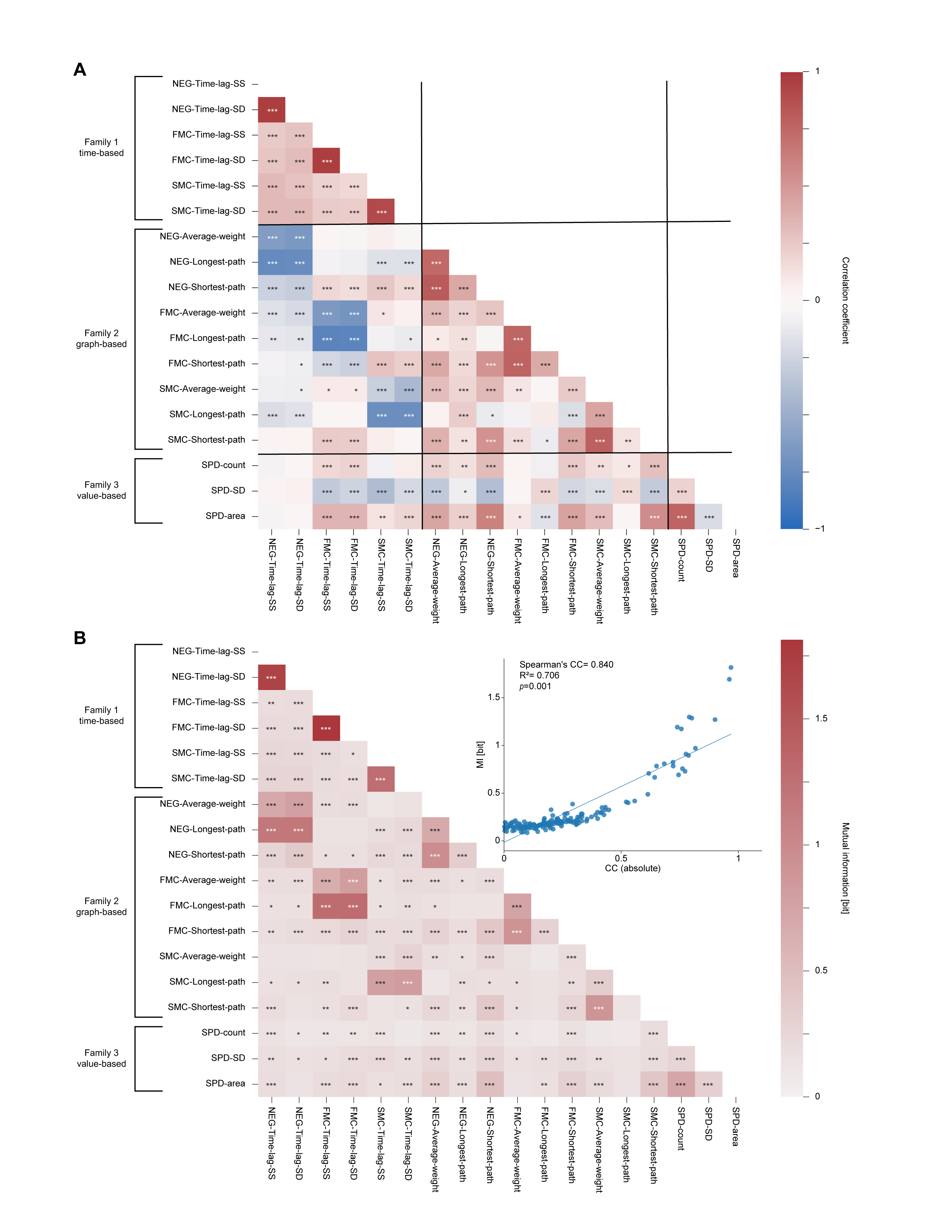

Supplement: Figure 3-1 — Extended Data for Figure 3. Spatial feature interrelations. A, Correlations between the spatial features, grouped by families. Eighty percent of the feature pairs (122 of 153) exhibit correlations that differ from zero. All conventions here and in B are the same as in Extended Data Figure 2-1. B, MI between spatial features. Most pairs (126 of 153; 82%) exhibit MI values that are higher than chance level. Inset, Scatter plot of the MI and the absolute CCs from A. Download Figure 3-1, TIF file. [file enu-eN-CFN-0265-22-s04.tif]

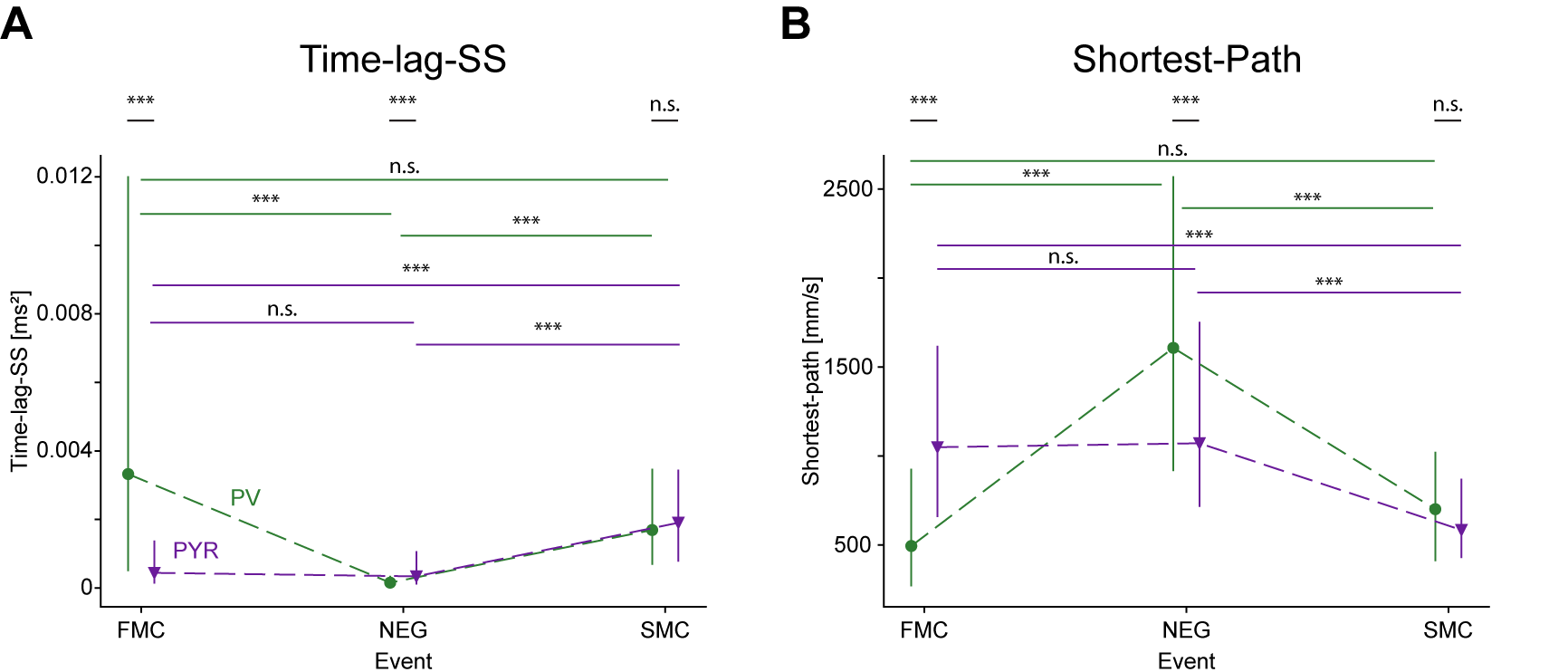

Supplement: Figure 4-1 — Extended Data for Figure 4. Time-lag-SS and Shortest-path features across events and cell types. A, Time-lag-SS features differ between events and cell types. Compared with PYR cells, PV cells show larger feature values during FMC. The relation reverses during the NEG event. The values during the SMC event are not consistently different between PYR and PV cells. For PV cells, feature values decrease from the FMC to the NEG, and then increase during the SMC. For PYR cells, feature values increase between FMC and SMC, and between NEG and SMC. Here and in B, all conventions are the same as in Figure 4A. B, The graph-based shortest-path feature differs between events and cell types. Compared with PYR cells, PV cells exhibit smaller feature values during FMC. The relation reverses during the NEG event. The values during the SMC event are not consistently different between PYR and PV cells. For PV cells, feature values increase from the FMC to the NEG, and then decrease during the SMC. For PYR cells, feature values decrease between FMC and SMC, and between NEG and SMC. Download Figure 4-1, TIF file. [file enu-eN-CFN-0265-22-s05.tif]

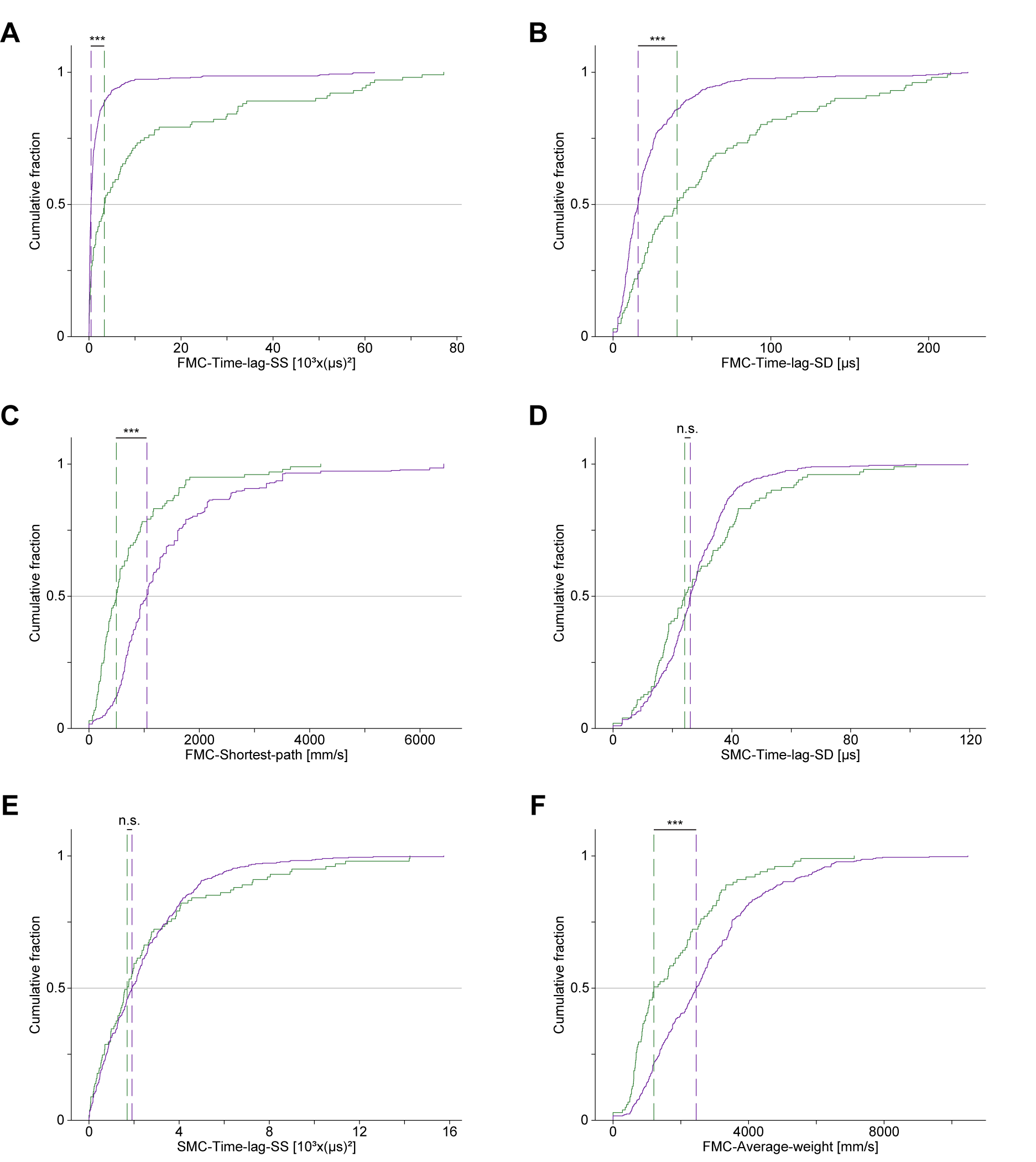

Supplement: Figure 5-1 — Extended Data for Figure 5. Distribution of the six most important spatial features. A, Cumulative distributions of the FMC-Time-lag-SS feature calculated without chunking. Here and in all subsequent cumulative distribution functions, horizontal lines represent 50%, vertical dashed lines indicate medians. n.s.p > 0.05; ***p < 0.001, U test. B, Cumulative distributions of the FMC-Time-lag-SD feature. C, Cumulative distributions of the FMC-Shortest-path feature. D, Cumulative distributions of the SMC-Time-lag-SD feature. E, Cumulative distributions of the SMC-Time-lag-SS feature. F, Cumulative distributions of the FMC-Average-weight feature. Download Figure 5-1, TIF file. [file enu-eN-CFN-0265-22-s06.tif]

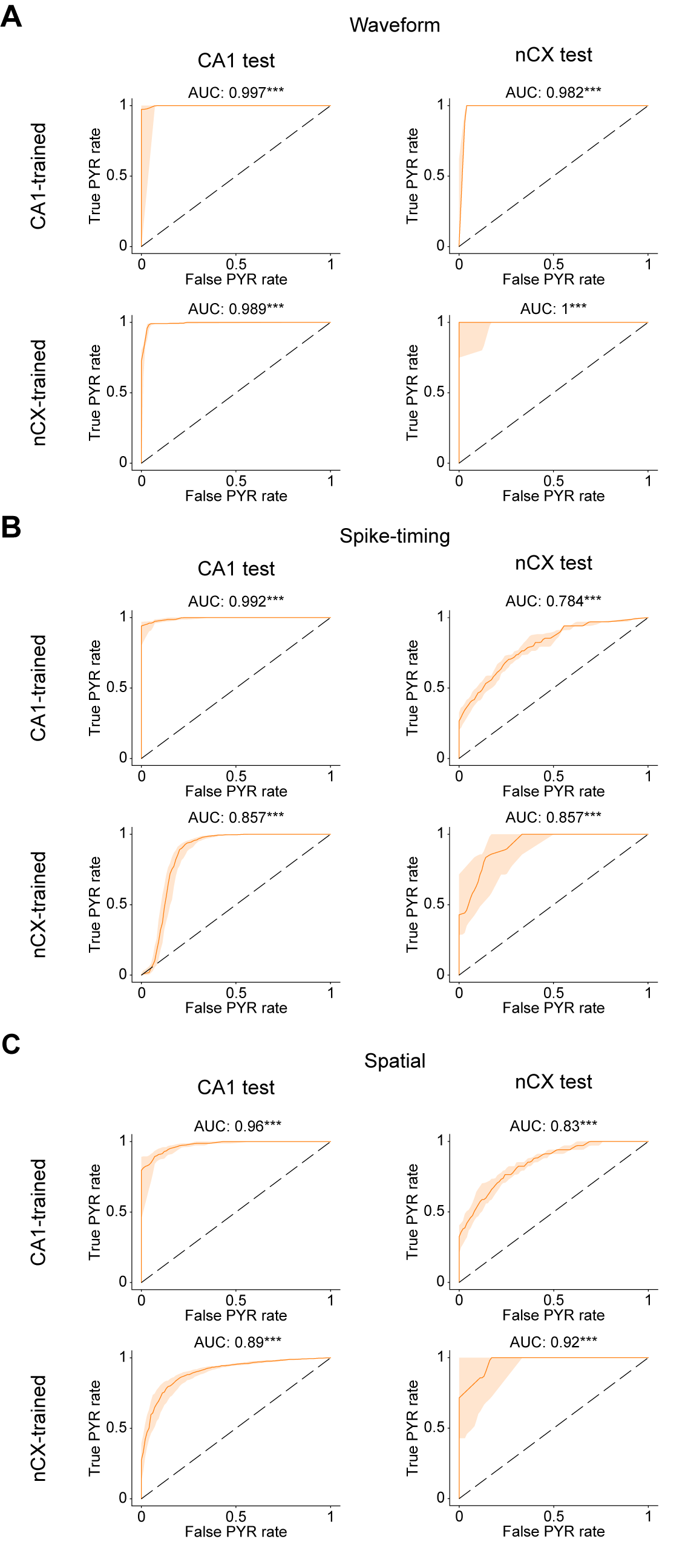

Supplement: Figure 6-1 — Extended Data for Figure 6. Models of all modalities generalize between brain regions. A, Models based on waveform-based features (50 spike chunks) were trained on CA1 data (top) or on neocortical data (bottom). The AUCs were calculated based on n = 50 models. Performance of waveform-based models is above chance level when tested on either CA1 (left) or neocortical (right) samples. Here and in B and C, ***p < 0.001, Wilcoxon test. B, Models based on spike-timing features (1600 spike chunks) were trained on CA1 data (top) or on neocortical data (bottom). Performance of spike-timing models is above chance level when tested on either CA1 or neocortical samples. C, Models based on spatial features (25 spike chunks) were trained on CA1 data (top) or on neocortical data (bottom). Performance of spatial models is above chance level when tested on either CA1 or neocortical samples. Download Figure 6-1, TIF file. [file enu-eN-CFN-0265-22-s07.tif]

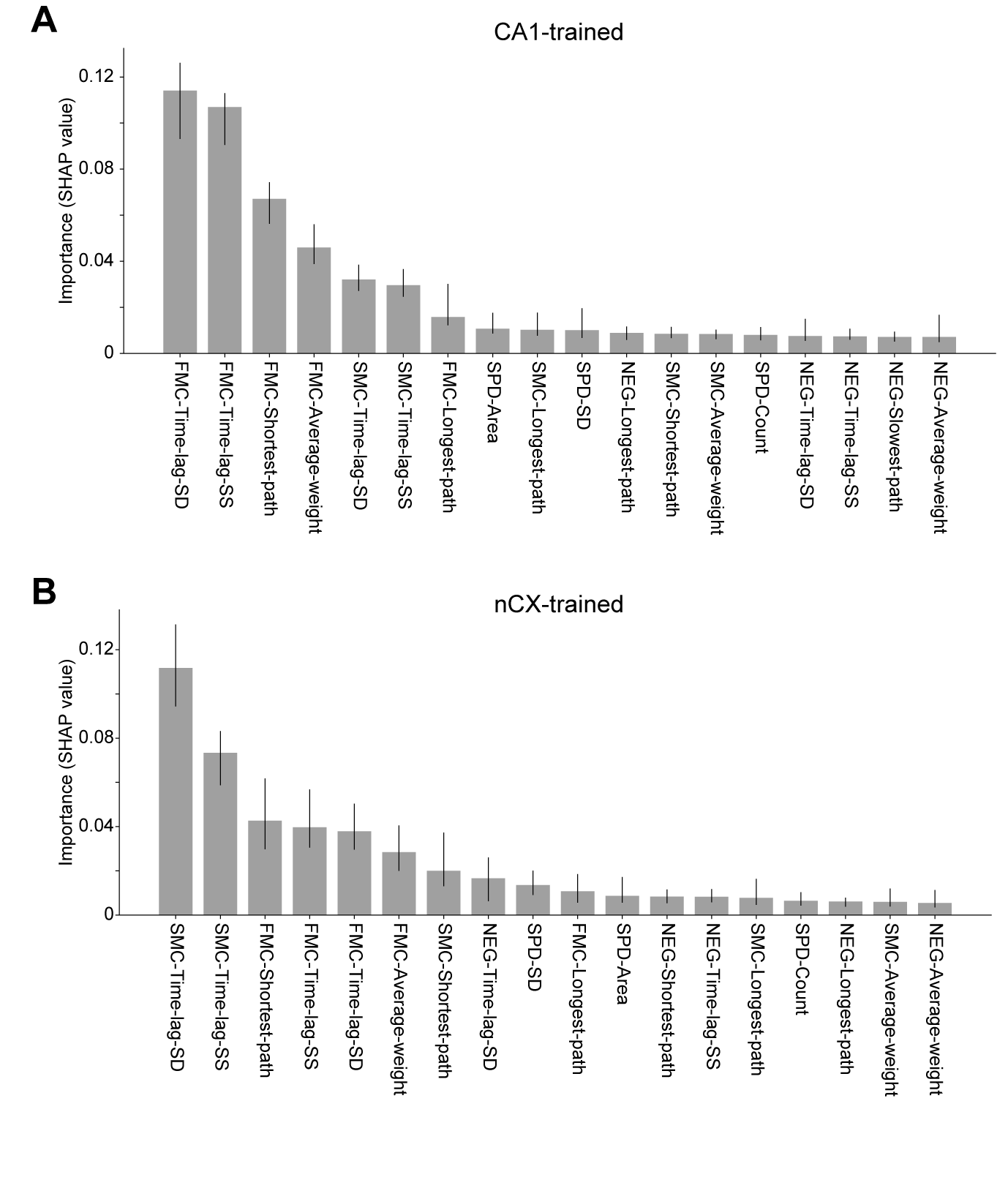

Supplement: Figure 6-2 — Extended Data for Figure 6. Spatial feature importance indicates consistent characteristics across regions. A, SHAP values for the spatial models with 25 spike chunks trained only on CA1 data. The six most important features are the same as the six most important features in the analyses of models trained on the data from both regions (Fig. 5C). B, SHAP values for the spatial models with 25 spike chunks trained on nCX data. The SMC features are the strongest determinants of the predictions for neocortical-trained models. The six most important features are the same as for the CA1-trained data (A) and as for the models trained on the data from both regions (Fig. 5C). Download Figure 6-2, TIF file. [file enu-eN-CFN-0265-22-s08.tif]
